# Data-Driven Cutoff Selection for the Patient Health Questionnaire-9 Depression Screening Tool

**DOI:** 10.1001/jamanetworkopen.2024.29630

**Published:** 2024-11-22

**Authors:** Brooke Levis, Parash Mani Bhandari, Dipika Neupane, Suiqiong Fan, Ying Sun, Chen He, Yin Wu, Ankur Krishnan, Zelalem Negeri, Mahrukh Imran, Danielle B. Rice, Kira E. Riehm, Marleine Azar, Alexander W. Levis, Jill Boruff, Pim Cuijpers, Simon Gilbody, John P. A. Ioannidis, Lorie A. Kloda, Scott B. Patten, Roy C. Ziegelstein, Daphna Harel, Yemisi Takwoingi, Sarah Markham, Sultan H. Alamri, Dagmar Amtmann, Bruce Arroll, Liat Ayalon, Hamid R. Baradaran, Anna Beraldi, Charles N. Bernstein, Arvin Bhana, Charles H. Bombardier, Ryna Imma Buji, Peter Butterworth, Gregory Carter, Marcos H. Chagas, Juliana C. N. Chan, Lai Fong Chan, Dixon Chibanda, Kerrie Clover, Aaron Conway, Yeates Conwell, Federico M. Daray, Janneke M. de Man-van Ginkel, Jesse R. Fann, Felix H. Fischer, Sally Field, Jane R. W. Fisher, Daniel S. S. Fung, Bizu Gelaye, Leila Gholizadeh, Felicity Goodyear-Smith, Eric P. Green, Catherine G. Greeno, Brian J. Hall, Liisa Hantsoo, Martin Härter, Leanne Hides, Stevan E. Hobfoll, Simone Honikman, Thomas Hyphantis, Masatoshi Inagaki, Maria Iglesias-Gonzalez, Hong Jin Jeon, Nathalie Jetté, Mohammad E. Khamseh, Kim M. Kiely, Brandon A. Kohrt, Yunxin Kwan, Maria Asunción Lara, Holly F. Levin-Aspenson, Shen-Ing Liu, Manote Lotrakul, Sonia R. Loureiro, Bernd Löwe, Nagendra P. Luitel, Crick Lund, Ruth Ann Marrie, Laura Marsh, Brian P. Marx, Anthony McGuire, Sherina Mohd Sidik, Tiago N. Munhoz, Kumiko Muramatsu, Juliet E. M. Nakku, Laura Navarrete, Flávia L. Osório, Brian W. Pence, Philippe Persoons, Inge Petersen, Angelo Picardi, Stephanie L. Pugh, Terence J. Quinn, Elmars Rancans, Sujit D. Rathod, Katrin Reuter, Alasdair G. Rooney, Iná S. Santos, Miranda T. Schram, Juwita Shaaban, Eileen H. Shinn, Abbey Sidebottom, Adam Simning, Lena Spangenberg, Lesley Stafford, Sharon C. Sung, Keiko Suzuki, Pei Lin Lynnette Tan, Martin Taylor-Rowan, Thach D. Tran, Alyna Turner, Christina M. van der Feltz-Cornelis, Thandi van Heyningen, Paul A. Vöhringer, Lynne I. Wagner, Jian Li Wang, David Watson, Jennifer White, Mary A. Whooley, Kirsty Winkley, Karen Wynter, Mitsuhiko Yamada, Qing Zhi Zeng, Yuying Zhang, Brett D. Thombs, Andrea Benedetti

**Affiliations:** 1 Lady Davis Institute for Medical Research, Jewish General Hospital, Montréal, Québec, Canada; 2 Department of Epidemiology, Biostatistics and Occupational Health, McGill University, Montréal, Québec, Canada; 3 Department of Statistics and Actuarial Science, University of Waterloo, Waterloo, Ontario, Canada; 4 Department of Psychiatry and Behavioural Neurosciences, McMaster University, Hamilton, Ontario, Canada; 5 Schulich Library of Physical Sciences, Life Sciences, and Engineering, McGill University, Montréal, Québec, Canada; 6 Department of Clinical, Neuro and Developmental Psychology, Amsterdam Public Health Research Institute, Vrije Universiteit Amsterdam, Amsterdam, the Netherlands; 7 Hull York Medical School and the Department of Health Sciences, University of York, Heslington, York, UK; 8 Department of Medicine, Stanford University, Stanford, California; 9 Department of Epidemiology and Population Health, Stanford University, Stanford, California; 10 Department of Biomedical Data Science,Stanford University, Stanford, California; 11 Department of Statistics, Stanford University, Stanford, California; 12 McGill University Libraries, Montréal, Québec, Canada; 13 Department of Community Health Sciences, University of Calgary, Calgary, Alberta, Canada; 14 Department of Medicine, Johns Hopkins University School of Medicine, Baltimore, Maryland; 15 Department of Applied Statistics, Social Science, and Humanities, New York University, New York; 16 Department of Applied Health Sciences, School of Health Sciences, College of Medicine and Health, University of Birmingham, Birmingham, UK; 17 Department of Biostatistics and Health Informatics, King's College London, London, UK; 18 Faculty of Medicine, King Abdulaziz University, Jeddah, Saudi Arabia; 19 Department of Rehabilitation Medicine, University of Washington, Seattle; 20 Department of General Practice and Primary Health Care, University of Auckland, Auckland, New Zealand; 21 Louis and Gabi Weisfeld School of Social Work, Bar Ilan University, Ramat Gan, Israel; 22 Endocrine Research Center, Institute of Endocrinology and Metabolism, Iran University of Medical Sciences, Tehran, Iran; 23 Kbo-Lech-Mangfall-Klinik Garmisch-Partenkirchen, Klinik für Psychiatrie, Psychotherapie and Psychosomatik, Lehrkrankenhaus der Technischen Universität München, Munich, Germany; 24 University of Manitoba IBD Clinical and Research Centre, Winnipeg, Manitoba, Canada; 25 Centre for Rural Health, School of Nursing and Public Health, College of Health Sciences, University of KwaZulu-Natal, Durban, KwaZulu-Natal, South Africa; 26 Department of Psychiatry, Hospital Mesra Bukit Padang, Sabah, Malaysia; 27 Centre for Epidemiology and Population Health, The Australian National University, Canberra, Australia; 28 Centre for Brain and Mental Health Research, University of Newcastle, Newcastle, New South Wales, Australia; 29 Department of Neurosciences and Behavior, Ribeirão Preto Medical School, University of São Paulo, Ribeirão Preto, São Paulo, Brazil; 30 Department of Medicine and Therapeutics, Hong Kong Institute of Diabetes and Obesity and Li Ka Shing Institute of Health Science, The Chinese University of Hong Kong, Prince of Wales Hospital, Shatin, Hong Kong Special Administrative Region, China; 31 Department of Psychiatry, National University of Malaysia, Kuala Lumpur, Malaysia; 32 Department of Community Medicine, University of Zimbabwe, Harare, Zimbabwe; 33 School of Medicine and Public Health, University of Newcastle, Callaghan, New South Wales, Australia; 34 School of Nursing, Queensland University of Technology, Brisbane, Queensland, Australia; 35 Department of Psychiatry, University of Rochester Medical Center, Rochester, New York; 36 Institute of Pharmacology, School of Medicine, University of Buenos Aires, Buenos Aires, Argentina; 37 Leids University Medical Center, Leiden, the Netherlands; 38 Department of Psychiatry and Behavioral Sciences, University of Washington, Seattle; 39 Center for Patient-Centered Outcomes Research, Department of Psychosomatic Medicine, Center for Internal Medicine and Dermatology, Charité - Universitätsmedizin Berlin, corporate member of Freie Universität Berlin, Humboldt-Universität zu Berlin, and Berlin Institute of Health, Berlin, Germany; 40 Perinatal Mental Health Project, Alan J Flisher Centre for Public Mental Health, Department of Psychiatry and Mental Health, University of Cape Town, Cape Town, South Africa; 41 Global and Women’s Health, Public Health and Preventive Medicine, Monash University, Melbourne, Victoria, Australia; 42 Department of Developmental Psychiatry, Institute of Mental Health, Singapore; 43 Department of Epidemiology, Harvard T.H. Chan School of Public Health, Boston, Massachusetts; 44 Faculty of Health, University of Technology Sydney, Sydney, New South Wales, Australia; 45 Department of General Practice and Primary Health Care, University of Auckland, Auckland, New Zealand; 46 Duke Global Health Institute, Duke University, Durham, North Carolina; 47 School of Social Work, University of Pittsburgh, Pittsburgh, Pennsylvania; 48 Center for Global Health Equity, New York University Shanghai, Shanghai, China; 49 Department of Psychiatry and Behavioral Sciences, The Johns Hopkins University School of Medicine, Baltimore, Maryland; 50 Department of Medical Psychology, University Medical Center Hamburg-Eppendorf, Hamburg, Germany; 51 School of Psychology, The University of Queensland, Brisbane, Queensland, Australia; 52 STAR–Stress, Anxiety and Resilience Consultants, Chicago, Illinois; 53 Department of Psychiatry, Faculty of Medicine, School of Health Sciences, University of Ioannina, Ioannina, Greece; 54 Department of Psychiatry, Faculty of Medicine, Shimane University, Izumo, Shimane, Japan; 55 Department of Psychiatry, Hospital Universitari Germans Trias i Pujol, Badalona, Spain; 56 Department of Psychiatry, Depression Center, Samsung Medical Center, Sungkyunkwan University School of Medicine, Seoul, South Korea; 57 Department of Clinical Neurosciences, University of Calgary, Calgary, Alberta, Canada; 58 Endocrine Research Center, Institute of Endocrinology and Metabolism, Iran University of Medical Sciences, Tehran, Iran; 59 School of Health and Society and School of Mathematics and Applied Statistics, University of Wollongong, Wollongong, New South Wales, Australia; 60 Center for Global Mental Health Equity, The George Washington University, Washington, DC; 61 Department of Psychological Medicine, Tan Tock Seng Hospital, Singapore; 62 Instituto Nacional de Psiquiatría Ramón de la Fuente Muñiz, San Lorenzo Huipulco, Tlalpan, Mexico; 63 Department of Psychology, University of North Texas, Denton; 64 Programme in Health Services and Systems Research, Duke-NUS Medical School, Singapore; 65 Department of Psychiatry, Faculty of Medicine, Ramathibodi Hospital, Mahidol University, Bangkok, Thailand; 66 Department of Neurosciences and Behavior, Ribeirão Preto Medical School, University of São Paulo, Ribeirão Preto, São Paulo, Brazil; 67 Department of Psychosomatic Medicine and Psychotherapy, University Medical Center Hamburg-Eppendorf, Hamburg, Germany; 68 Research Department, TPO Nepal, Kathmandu, Nepal; 69 Centre for Global Mental Health, Health Service and Population Research Department, Institute of Psychiatry, Psychology and Neuroscience, King’s College London, London, UK; 70 Departments of Medicine and Community Health Sciences, Max Rady College of Medicine, Rady Faculty of Health Sciences, University of Manitoba, Winnipeg, Manitoba, Canada; 71 Baylor College of Medicine, Houston and Michael E. DeBakey Veterans Affairs Medical Center, Houston, Texas; 72 National Center for PTSD at Veterans Affairs Boston Healthcare System, Boston, Massachusetts; 73 College of Nursing, University of South Florida, Tampa; 74 Department of Psychiatry, Faculty of Medicine and Health Sciences, Universiti Putra Malaysia, Serdang, Selangor, Malaysia; 75 Post-Graduate Program in Epidemiology, Federal University of Pelotas, Pelotas, Rio Grande do Sul, Brazil; 76 Niigata Seiryo University Health Service Center, Niigata, Japan; 77 Butabika National Referral Teaching Hospital, Kampala, Uganda; 78 Department of Epidemiology and Psychosocial Research, Instituto Nacional de Psiquiatría Ramón de la Fuente Muñiz, Ciudad de México, México; 79 Department of Epidemiology, Gillings School of Global Public Health, The University of North Carolina at Chapel Hill, Chapel Hill; 80 Department of Psycho-Pedagogic Psychiatry, Healthcare Group Sint-Kamillus, Broeders van Liefde, Bierbeek, Belgium; 81 Centre for Behavioural Sciences and Mental Health, Italian National Institute of Health, Rome, Italy; 82 Department of Statistics, American College of Radiology, NRG Oncology Statistics and Data Management Center, Philadelphia, Pennsylvania; 83 Institute of Cardiovascular and Medical Sciences, University of Glasgow, Glasgow, Scotland, UK; 84 Department of Psychiatry and Narcology, Riga Stradins University, Riga, Latvia; 85 Department of Population Health, London School of Hygiene and Tropical Medicine, London, UK; 86 Group Practice for Psychotherapy and Psycho-oncology, Freiburg, Germany; 87 Division of Psychiatry, Royal Edinburgh Hospital, The University of Edinburgh, Edinburgh, Scotland, UK; 88 Department of Internal Medicine, Maastricht University Medical Center, Maastricht, the Netherlands; 89 Department of Family Medicine, School of Medical Sciences, Universiti Sains Malaysia, Kelantan, Malaysia; 90 Department of Behavioral Science, The University of Texas M.D. Anderson Cancer Center, Houston; 91 Allina Health, Minneapolis, Minnesota; 92 Department of Medical Psychology and Medical Sociology, University of Leipzig, Leipzig, Germany; 93 Melbourne School of Psychological Sciences, The University of Melbourne, Melbourne, Victoria, Australia; 94 Department of General Medicine, Asahikawa University Hospital, Asahikawa, Hokkaido, Japan; 95 Institute of Health and Wellbeing, University of Glasgow, Glasgow, Scotland, UK; 96 IMPACT–the Institute for Mental and Physical Health and Clinical Translation, School of Medicine, Deakin University, Geelong, Victoria, Australia; 97 Department of Health Sciences, Hull York Medical School, University of York, York, UK; 98 Justice and Violence Prevention Programme, Institute for Security Studies, Pretoria, South Africa; 99 Department of Psychiatry and Mental Health, Clinical Hospital, Universidad de Chile, Santiago, Chile; 100 Department of Health Policy and Management, Gillings School of Global Public Health, The University of North Carolina at Chapel Hill, Chapel Hill; 101 Department of Community Health and Epidemiology, Faculty of Medicine, Dalhousie University, Halifax, Nova Scotia, Canada; 102 Department of Psychology, University of Notre Dame, Notre Dame, Indiana; 103 School of Medicine and Public Health, College of Health, Medicine and Wellbeing, University of Newcastle, New South Wales, Australia; 104 Department of Medicine, University of California San Francisco, San Francisco; 105 Department of Epidemiology and Biostatistics, University of California San Francisco, San Francisco; 106 Florence Nightingale Faculty of Nursing, Midwifery and Palliative Care, King's College London, London, UK; 107 School of Clinical Sciences, Monash University, Melbourne, Victoria, Australia; 108 Department of Pathophysiology, Tokyo Kasei Gakuin University, Chiyoda-ku, Tokyo, Japan; 109 Shanghai Mental Health Center, Shanghai Jiao Tong University School of Medicine, Shanghai, China; 110 Department of Medicine and Therapeutics, Prince of Wales Hospital, The Chinese University of Hong Kong, Hong Kong Special Administrative Region, China; 111 Department of Psychiatry, McGill University, Montréal, Québec, Canada; 112 Department of Medicine, McGill University, Montréal, Québec, Canada; 113 Department of Psychology, McGill University, Montréal, Québec, Canada; 114 Biomedical Ethics Unit, McGill University, Montréal, Québec, Canada; 115 Respiratory Epidemiology and Clinical Research Unit, McGill University Health Centre, Montréal, Québec, Canada; 116 Centre for Outcomes Research and Evaluation, Research Institute of the McGill University Health Centre, Montréal, Québec, Canada

## Abstract

**Importance:**

Test accuracy studies often use small datasets to simultaneously select an optimal cutoff score that maximizes test accuracy and generate accuracy estimates.

**Objective:**

To evaluate the degree to which using data-driven methods to simultaneously select an optimal Patient Health Questionnaire-9 (PHQ-9) cutoff score and estimate accuracy yields (1) optimal cutoff scores that differ from the population-level optimal cutoff score and (2) biased accuracy estimates.

**Design, Setting, and Participants:**

This study used cross-sectional data from an existing individual participant data meta-analysis (IPDMA) database on PHQ-9 screening accuracy to represent a hypothetical population. Studies in the IPDMA database compared participant PHQ-9 scores with a major depression classification. From the IPDMA population, 1000 studies of 100, 200, 500, and 1000 participants each were resampled.

**Main Outcomes and Measures:**

For the full IPDMA population and each simulated study, an optimal cutoff score was selected by maximizing the Youden index. Accuracy estimates for optimal cutoff scores in simulated studies were compared with accuracy in the full population.

**Results:**

The IPDMA database included 100 primary studies with 44 503 participants (4541 [10%] cases of major depression). The population-level optimal cutoff score was 8 or higher. Optimal cutoff scores in simulated studies ranged from 2 or higher to 21 or higher in samples of 100 participants and 5 or higher to 11 or higher in samples of 1000 participants. The percentage of simulated studies that identified the true optimal cutoff score of 8 or higher was 17% for samples of 100 participants and 33% for samples of 1000 participants. Compared with estimates for a cutoff score of 8 or higher in the population, sensitivity was overestimated by 6.4 (95% CI, 5.7-7.1) percentage points in samples of 100 participants, 4.9 (95% CI, 4.3-5.5) percentage points in samples of 200 participants, 2.2 (95% CI, 1.8-2.6) percentage points in samples of 500 participants, and 1.8 (95% CI, 1.5-2.1) percentage points in samples of 1000 participants. Specificity was within 1 percentage point across sample sizes.

**Conclusions and Relevance:**

This study of cross-sectional data found that optimal cutoff scores and accuracy estimates differed substantially from population values when data-driven methods were used to simultaneously identify an optimal cutoff score and estimate accuracy. Users of diagnostic accuracy evidence should evaluate studies of accuracy with caution and ensure that cutoff score recommendations are based on adequately powered research or well-conducted meta-analyses.

## Key Points

**Question:**

Does data-driven optimal cutoff score selection in Patient Health Questionnaire-9 (PHQ-9) screening accuracy studies generate cutoff scores that diverge from the population-level cutoff score and overstate accuracy?

**Findings:**

In this study of cross-sectional data from 100 primary studies including 44 503 participants, the optimal PHQ-9 scores identified varied from the population-level optimal cutoff score, and PHQ-9 screening accuracy was exaggerated. As sample size increased, overestimation of sensitivity decreased, while specificity remained within 1 percentage point.

**Meaning:**

Findings of this study suggest that users of diagnostic accuracy evidence should evaluate studies of accuracy with caution and ensure that cutoff score recommendations are based on adequately powered research or well-conducted meta-analyses.

## Teaser

This study of cross-sectional data evaluates the cutoff selection process and sensitivity and specificity of accuracy estimation in Patient Health Questionnaire-9 screening accuracy studies.

## Introduction

Studies on depression screening tool accuracy often use data-driven approaches and small samples and numbers of depression cases to simultaneously establish an optimal cutoff score and estimate accuracy.^1,2,3^ A recent review of 172 studies found a median sample size of 194 and median number of depression cases of approximately 20.^1^ Seventy-six percent of the included studies identified an optimal cutoff score that diverged from a standard cutoff score, and authors of 40% of those studies recommended using their optimal cutoff score, rather than the standard cutoff, in their population.^1^

Previous studies on data-driven selection of test cutoff scores have reported that these methods produce overly optimistic accuracy estimates, especially in small samples.^4,5,6,7,8^ However, most of these studies used simulated datasets based on hypothetical test score distributions rather than real participant data. A previous study analyzed Edinburgh Postnatal Depression Scale (EPDS) data for 13 255 participants and found that in 1000 simulated or resampled studies, the cutoff score maximizing the Youden index (sensitivity + specificity – 1)^9^ ranged from 5 or higher to 17 or higher with resampled studies of 100 participants and from 8 or higher to 13 or higher with 1000 participants.^8^ Mean sensitivity overestimation was 7 percentage points for 100 participants vs 1 percentage point for resampled studies of 1000 participants, while specificity was underestimated by 1 percentage point across sample sizes.^8^

The standard cutoff score traditionally used to screen for major depression with the Patient Health Questionnaire-9 (PHQ-9) is 10 or higher.^10,11,12,13,14^ An individual participant data meta-analysis (IPDMA) of 100 primary studies (44 503 participants and 4541 cases of major depression) confirmed that a cutoff score of 10 or higher maximized combined sensitivity and specificity in studies that used a gold standard semistructured interview reference standard, although the optimal cutoff score was 8 or higher when fully structured interviews designed for lay administration were used.^15,16^

Many primary studies of PHQ-9 accuracy emphasize results from data-driven optimal cutoff scores.^1^ The degree to which accuracy is overestimated when data-driven cutoff scores are used for the PHQ-9, however, is not known. The objective of this study was to evaluate the degree to which using data-driven methods to simultaneously select an optimal PHQ-9 cutoff score and estimate accuracy yields biased estimates. We estimated, across different sample sizes, the degree to which data-driven cutoff score selection was a factor in (1) sample-specific optimal cutoff scores that differed from the population-level optimal cutoff score and (2) biased accuracy estimates. For comparison, we also estimated accuracy using the population-level optimal cutoff score in individual resampled studies and compared them with population accuracy.

## Methods

The Jewish General Hospital Research Ethics Committee deemed this study of cross-sectional data exempt from ethics approval and the informed consent requirement since the study involved IPDMA of previously collected deidentified data. For each included dataset, we confirmed that the original study received ethics approval and the participants provided informed consent. We followed the Strengthening the Reporting of Observational Studies in Epidemiology (STROBE) reporting guideline.

We used data from an IPDMA of PHQ-9 diagnostic accuracy (hereafter, main IPDMA) to represent a hypothetical population from which studies of different sizes could be resampled.^16^ Data in the IPDMA database were identified from a literature search covering January 1, 2000, through May 9, 2018. The main IPDMA was registered in PROSPERO (CRD42014010673), and a protocol was published.^17^ A protocol for the present study was published in the Open Science Framework repository prior to initiation.^18^ Details on the methods used to identify, obtain, and synthesize the data included in the present study are provided in eMethods 1 and 2 in Supplement 1. We used a similar methodological approach as that in the previous EPDS resampling study.^8^ Because of the overlap of methods in the present study and previous studies, we described the methods similarly and followed the reporting guidance from the Text Recycling Research Project.^19^

### Statistical Analysis

For the purposes of the present study, we used the main IPDMA dataset to represent a hypothetical population and defined population sensitivity and specificity values for PHQ-9 cutoff scores to be those estimated in the hypothetical population. In the main IPDMA, we accounted for clustering of observations within each study, and we applied sampling weights to account for imbalances in participant samples when, for instance, all participants with positive PHQ-9 results but only a random portion of those with negative PHQ-9 results were administered a diagnostic interview. In the present study, we ignored clustering and sampling weights to have a defined population from which we could draw samples that represented simulated primary studies and to be able to analyze the population data and simulated primary study data with the same analytical approach. In addition, in the main IPDMA, we stratified included studies by reference standard type because previous studies have shown that different types of diagnostic interviews classify major depression differently.^20,21^ However, in primary analyses of the present study, we did not stratify the studies by reference standard because we were not evaluating the true screening accuracy of the PHQ-9, and combining included studies that used different reference standards allowed us to have a single hypothetical population for resampling. As a result, this procedure produced accuracy estimates that differed from those reported in the main IPDMA.^16^ In the present study, we calculated the population-level optimal cutoff score that maximized the Youden index in the full IPDMA dataset, which was 8 or higher.

First, we described the individual primary studies included in the main IPDMA dataset in terms of sample size, number of major depression cases, and optimal cutoff score (based on maximizing the Youden index). If there was a tie in maximum Youden index between multiple cutoff scores, we randomly selected 1 of the cutoff scores. We used the Youden index because it is by far the most common method for selecting optimal cutoff scores in depression screening accuracy studies, and our study aimed to reflect current research practices.^1^

Second, from the main IPDMA dataset, we sampled with replacement to generate 1000 randomly sampled studies with 100, 200, 500, and 1000 participants each to mimic what would occur in primary studies that use samples of these sizes. For each study, we defined the sample-specific optimal cutoff score as the cutoff that maximized the Youden index, with random selection in case of ties. For each sample size across the 1000 samples, we (1) graphically illustrated the variability in sample-specific optimal cutoff scores and their accuracy estimates and (2) calculated the mean difference in sensitivity and specificity estimates at the sample-specific optimal cutoff scores and at a cutoff score of 8 or higher compared with sensitivity and specificity estimates for a cutoff score of 8 or higher in the population. In additional analyses, we stratified results by optimal cutoff value.

Random selection of participants in simulated samples and averaging sensitivity and specificity across 1000 samples for each sample size were performed to balance other possible sources of divergent accuracy, such as reference standards or individual participant characteristics. Nonetheless, in sensitivity analyses, we repeated the resampling process, including only studies that used the semistructured Structured Clinical Interview for *DSM* (*Diagnostic and Statistical Manual of Mental Disorders*) Disorders as the reference standard.

For all analyses, sensitivity and specificity were estimated using 2 × 2 table counts. Analyses were performed using R, version 4.2.2 (R Project for Statistical Computing).

## Results

The full IPDMA database included 100 primary studies with 44 503 participants (4541 cases [10%] of major depression), which constituted the population for the present study. eTable 1 in Supplement 1 provides the primary study characteristics. In the 100 included studies, the median (IQR) sample size was 194 (134-386) and the median (IQR) number of major depression cases was 28 (14-60). Study-specific optimal cutoff scores ranged from 3 or higher to 18 or higher (median, ≥10). Frequencies of PHQ-9 scores for cases and noncases are provided in eTable 2 in Supplement 1, with histograms in the eFigure in Supplement 1. The PHQ-9 scores were normally distributed among cases (mean [SD], 13 [6]; median [IQR], 13 [9-18]) and right-skewed among noncases (mean [SD], 4 [4]; median [IQR], 3 [1-6]). In the full IPDMA database population, unweighted sensitivity and specificity for PHQ-9 score of 8 or higher were 80.4% and 82.0%, respectively.

### Variability of Sample-Specific Optimal Cutoff Scores in Simulated Samples

**Figure 1.**
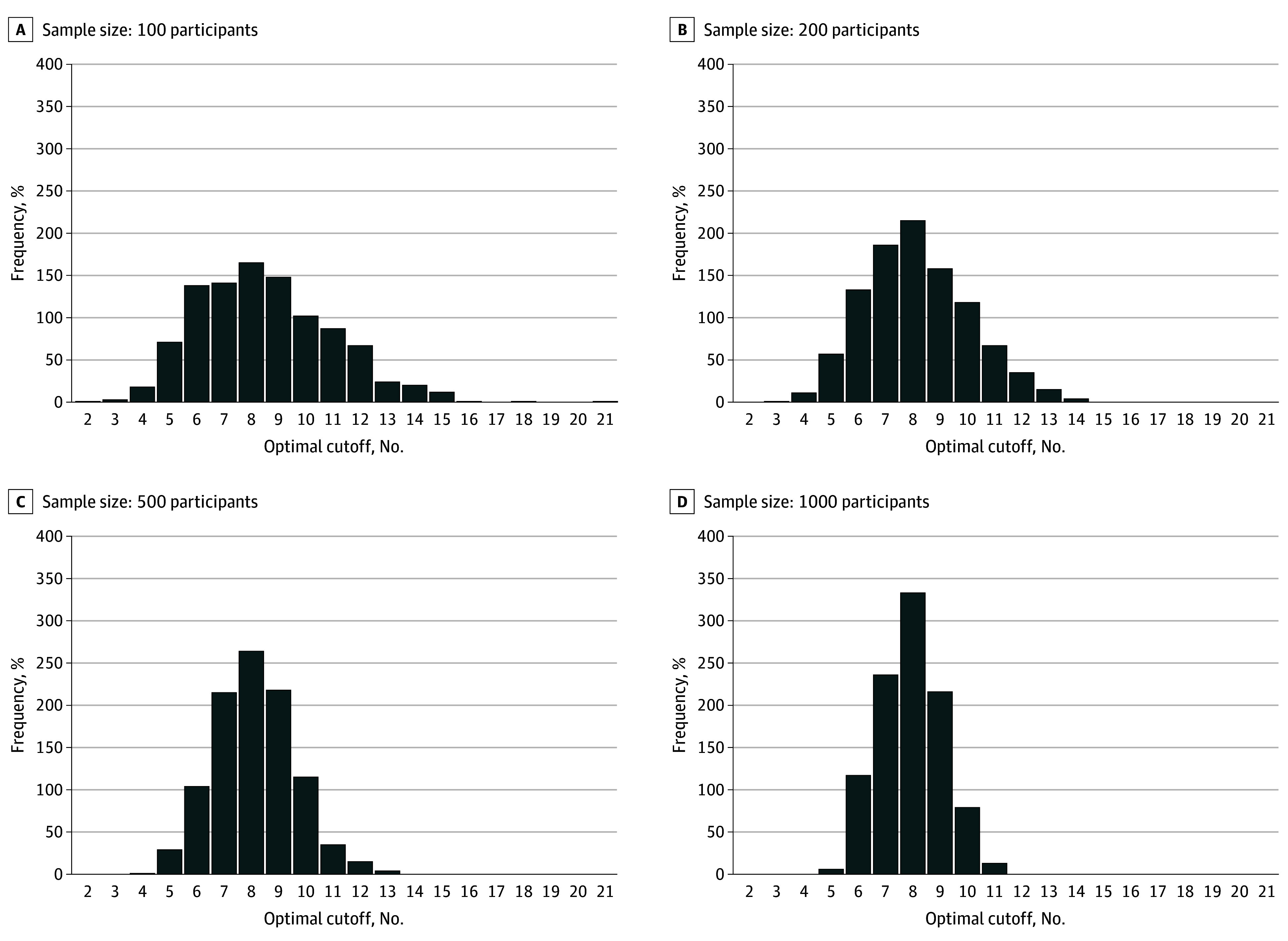
Variability of Data-Driven Optimal Cutoff Scores in 1000 Resampled Studies of 100, 200, 500, and 1000 Participants

Figure 1 shows the variability of sample-specific optimal cutoff scores from 1000 resampled studies of 100, 200, 500, and 1000 participants. As sample size increased, the variability in sample-specific optimal cutoff scores decreased. Of the 1000 resampled studies of 100 participants, study-specific optimal cutoff scores ranged from 2 or higher to 21 or higher; 17% of resampled studies had an optimal cutoff score of 8 or higher, and 45% of resampled studies had an optimal cutoff score between 7 or higher and 9 or higher. When sample size of the resampled studies increased to 1000 participants per study, the range of optimal cutoff scores was 5 or higher to 11 or higher; 33% of resampled studies had an optimal cutoff score of 8 or higher, and 79% of resampled studies had an optimal cutoff score between 7 or higher and 9 or higher.

### Bias and Sensitivity Analyses in Simulated Samples

**Figure 2.**
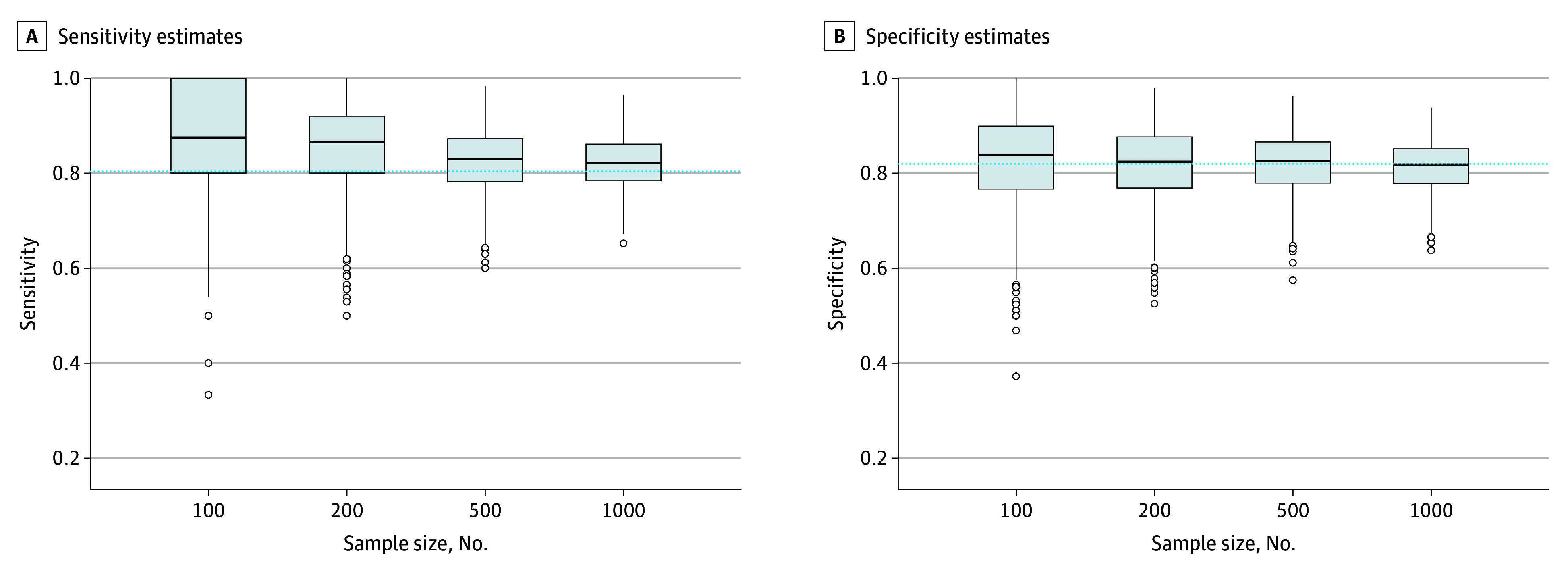
Variability in Accuracy Estimates of the Optimal Cutoff Scores in 1000 Resampled Studies of 100, 200, 500, and 1000 Participants vs Accuracy Values for a Cutoff of 8 or Higher in the Population Edges of boxes represent the 25th and 75th percentiles; horizontal line inside boxes represents the median; dashed horizontal line represents the accuracy of the true population-level optimal cutoff score in the full Patient Health Questionnaire-9 individual participant data meta-analysis dataset (cutoff score ≥8; sensitivity = 80.4%, specificity = 82.0%); and dots represent outliers.

**Figure 3.**
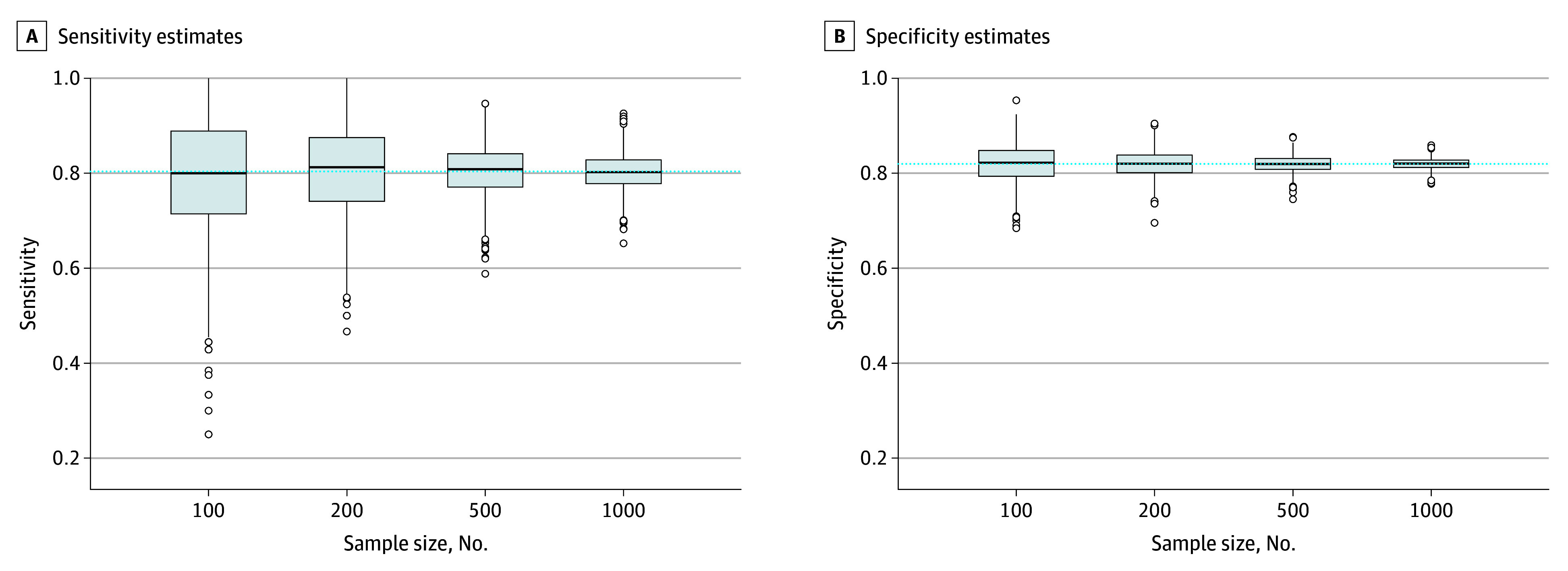
Variability in Accuracy Estimates of a Cutoff Score of 8 or Higher in 1000 Resampled Studies of 100, 200, 500, and 1000 Participants vs Accuracy Values for a Cutoff of 8 or Higher in the Population Edges of boxes represent the 25th and 75th percentiles; horizontal line inside boxes represents the median; dashed horizontal line represents the accuracy of the true population-level optimal cutoff score in the full Patient Health Questionnaire-9 individual participant data meta-analysis dataset (cutoff score ≥8; sensitivity = 80.4%, specificity = 82.0%); and dots represent outliers.

As shown in Figure 2, overestimation of sensitivity estimates for sample-specific optimal cutoff scores decreased with increasing sample size, whereas specificity estimates remained within 1 percentage point across sample sizes. Precision of both sensitivity and specificity estimates increased with sample size. As shown in the Table, compared with accuracy estimates for a cutoff score of 8 or higher in the full IPDMA database, study-specific optimal cutoff scores in samples of 100 participants overestimated sensitivity by a mean of 6.4 (95% CI, 5.7-7.1) percentage points and overestimated specificity by 0.6 (95% CI, 0.0-1.2) percentage points. In samples of 200 and 500 participants, sensitivity was overestimated by 4.9 (95% CI, 4.3-5.5) and 2.2 (95% CI, 1.8-2.6) percentage points, respectively, and specificity was underestimated by 0.3 percentage points (mean difference, –0.3 [95% CI, –0.8 to 0.2] percentage points) and 0.0 (95% CI, –0.4 to 0.3) percentage points, respectively. When sample size increased to 1000, study-specific optimal cutoff scores overestimated sensitivity by 1.8 (95% CI, 1.5-2.1) percentage points and underestimated specificity by 0.6 percentage points (mean difference, –0.6 [95% CI, −1.0 to −0.3] percentage points). As shown in the Table and Figure 3, when each resampled study used a prespecified cutoff score of 8 or higher, mean sample-specific sensitivity and specificity values were similar to those in the population for all sample sizes.

**Table.** Mean Bias of Accuracy Estimates for 1000 Resampled Studies of 100, 200, 500, and 1000 Participants

	Mean difference (95% CI), percentage points							
	Sample size = 100		Sample size = 200		Sample size = 500		Sample size = 1000	
	Sensitivity	Specificity	Sensitivity	Specificity	Sensitivity	Specificity	Sensitivity	Specificity
Sample-specific optimal cutoff score^a^ – Population-level optimal cutoff score ≥8^b^	6.4 (5.7 to 7.1)	0.6 (0.0 to 1.2)	4.9 (4.3 to 5.5)	−0.3 (−0.8 to 0.2)	2.2 (1.8 to 2.6)	0.0 (−0.4 to 0.3)	1.8 (1.5 to 2.1)	−0.6 (−1.0 to −0.3)
Sample-specific cutoff score ≥8 – Population-level optimal cutoff score ≥8	−0.8 (−1.7 to 0.0)	0.1 (−0.1 to 0.4)	0.2 (−0.3 to 0.8)	−0.1 (−0.2 to 0.1)	0.1 (−0.2 to 0.4)	0.0 (−0.1 to 0.1)	−0.1 (−0.4 to 0.1)	0.0 (−0.1 to 0.1)

^a^ Sample-specific optimal cutoff score refers to the cutoff score maximizing the Youden index in each simulated sample.

^b^ The optimal cutoff score in the full Patient Health Questionnaire-9 individual participant data meta-analysis dataset is 8 or higher (sensitivity = 80.4%, specificity = 82.0%).

As shown in eTable 3 in Supplement 1, across sample sizes, bias in estimates increased as the sample-specific optimal cutoff score diverged from 8 or higher. When the sample-specific optimal cutoff score was lower than 8, specificity was underestimated (by 6 percentage points for cutoff scores of 6 or 7 and by 16-17 percentage points for cutoff scores ≤5), whereas when the sample-specific optimal cutoff score was higher than 8, specificity was overestimated (by 5-6 percentage points for cutoff scores of 9 or 10 and by 9-11 percentage points for cutoff scores ≥11). The opposite pattern was seen for sensitivity, although there was a shift in values given that even when the sample-specific cutoff score was exactly 8 or higher, sensitivity was, on average, overestimated. As shown in eTables 4 and 5 in Supplement 1, variability in sample-specific optimal cutoff scores and bias in sensitivity and specificity were similar to the primary results when only studies that used the Structured Clinical Interview for *DSM* Disorders reference standard were included.

## Discussion

To our knowledge, this was the first study to assess bias in PHQ-9 accuracy estimates due to data-driven optimal cutoff score selection. The main finding of this study was that data-driven optimal PHQ-9 cutoff scores often differed from the population-level optimal cutoff score, sometimes substantially, and generated biased accuracy estimates. As sample size increased from 100 to 1000 participants, variability in optimal cutoff scores decreased from a range of 2 or higher to 21 or higher to a range of 5 or higher to 11 or higher, and overestimation in sensitivity compared with the population value decreased from 6.4 to 1.8 percentage points, while specificity remained within 1 percentage point. The magnitude and direction of bias differed depending on how far the sample-specific optimal cutoff score was from the population-level optimal cutoff score of 8 or higher. When a predefined cutoff score of 8 or higher was used in resampled studies, mean accuracy estimates were consistent with overall population estimates.

### Comparison With Other Studies

Previous distribution-based simulation studies have found that data-driven cutoff selection in small samples yields exaggerated accuracy estimates.^4,5,6,7^ Most studies on depression screening tool accuracy have small sample sizes and numbers of depression cases. Individual studies often report results from 1, several, or many cutoff scores such that there is a wide range of optimal cutoff scores and accuracy estimates across studies in the literature.^1,2,3^ Many researchers conclude that sample characteristics alter accuracy and that different optimal cutoff scores are needed for particular population subgroups. Results from the present study and the previous EPDS resampling study^8^ suggest that variability in optimal cutoff scores and accuracy estimates often occurs due to chance and imprecision in small samples even when all samples are drawn from the same population. The finding that data-driven methods and small samples may explain divergent results across studies is consistent with the results of several large IPDMA studies,^15,16,22,23^ which found that there were no substantive differences in depression screening tool accuracy based on participant characteristics. Additionally, the finding in the present study that accuracy estimates were similar between the full population and resampled studies when the same cutoff score was used underlines that divergences can be attributed to data-driven methods and sample size rather than to characteristics of participants in each sample.

The finding that there were larger biases in sensitivity than in specificity was not surprising given that most studies had many fewer participants with depression than without. In addition, PHQ-9 scores among cases were normally distributed, whereas scores among noncases were heavily right-skewed. Similar results were seen in the previous EPDS resampling study, which found that overestimation of sensitivity reduced from 7 percentage points in samples of 100 participants to 1 percentage point in samples of 1000 participants, while specificity was underestimated by 1 percentage point across sample sizes.^8^ These findings suggest that data-driven methods for cutoff score selection can allow for substantial sensitivity gains with only minor costs to specificity, although at the individual study level, sensitivity can be either overestimated or underestimated.

### Implications

Clinicians and policymakers who make decisions regarding depression screening should interpret cautiously the optimal cutoff scores for the PHQ-9 and other depression screening tools identified in small single studies. Ideally, the decisions regarding what cutoff scores to use should be based on large, well-conducted meta-analyses or on multiple validations in studies with adequate sample sizes for desired precision levels. In addition, clinicians may prioritize either sensitivity or specificity in different clinical settings rather than consider them equally, as is the practice when selecting cutoff scores based on the Youden index, and select higher or lower cutoff scores depending on health and resource priorities.^24^ The optimal cutoff score of 8 or higher, which was identified in the hypothetical population of this study, was not derived using methods that accounted for clustering and sample weights and may reflect that participants from studies that used different reference standards were combined. PHQ-9 cutoff scores and accuracy estimates from the main IPDMA should be used clinically.^16^ Depression screening questionnaires are not intended to establish clinical diagnoses but can be used for screening followed by clinical evaluation of those who receive positive results. Whether screening should occur in practice requires evidence from clinical trials of screening benefit, which has not been established.^25^

Although the Standards for Reporting of Diagnostic Accuracy Studies reporting guideline recommends a priori sample size calculations,^26^ most depression screening tool accuracy studies do not conduct such calculations.^2,3^ Researchers conducting primary studies on accuracy should conduct sample size calculations prior to recruitment to ensure the inclusion of sufficient numbers of both cases and noncases for desired precision levels in accuracy estimates.^27^ In addition, selective cutoff reporting bias occurs when researchers select the cutoff scores for which to report accuracy results in their individual studies based on the relative accuracy of those cutoff scores in their sample (eg, reporting accuracy estimates for cutoff scores that maximize the Youden index but not for other cutoff scores).^28,29^ Selective cutoff reporting bias has been found to underestimate sensitivity for cutoff scores below a clearly defined standard and overestimate sensitivity for cutoff scores above the standard.^28,29^ Since summary accuracy estimates for a predefined cutoff score do not tend to be biased, researchers should report accuracy estimates for all possible cutoff scores rather than just those that are optimal in a given study or close to the optimal cutoff score.^28,29^ Additionally, statistical methods for estimating cutoff scores and out-of-sample performance, such as smoothing based on kernel estimation and bootstrapping, should be considered.^30^

Beyond variability in accuracy estimates, researchers should also consider variability in the optimal cutoff score that may be identified in individual studies. It is possible that researchers could use statistical methods to estimate uncertainty around optimal cutoff scores in their individual studies (eg, via CIs^31,32^) and use internal validation methods (eg, bootstrapping) to adjust for bias due to optimism.^30,33^ Further work to test and demonstrate such methods for the purpose of mental health screening is needed.

### Strengths and Limitations

A study strength is the use of a large sample and real participant data. A limitation to consider is that we did not include datasets from recently published studies on PHQ-9 accuracy; however, we do not expect that the inclusion of more recent studies would alter the results given that newer studies would likely have similar sample sizes and heterogeneity. We included data from 100 primary studies, and we believe that the dataset used for the present study adequately represents a hypothetical population for resampling purposes. A second limitation is that we used only the Youden index to select optimal cutoff scores. Although it is by far the most common method used in depression screening accuracy studies^1^ and performs similarly to other indices (eg, the Euclidean distance),^34^ the Youden index is known to be unreliable and prone to overestimation. It is possible that results could differ slightly for an alternative method.

## Conclusions

Using samples with small numbers of participants and cases to simultaneously identify an optimal cutoff score and estimate its accuracy yielded optimal cutoff scores that varied widely from study to study and exaggerated accuracy estimates. Variability in optimal cutoff scores and the extent of sensitivity exaggeration decreased as sample size increased. Researchers should conduct a priori sample size calculations to ensure the inclusion of sufficient numbers of both cases and noncases in diagnostic accuracy studies, report accuracy estimates for all cutoff scores rather than only for study-specific optimal cutoff scores, and avoid making recommendations about optimal cutoff scores and accuracy based on small single studies. Researchers also should consider using statistical methods that improve optimal cutoff score identification and estimation of accuracy outside of the study sample. Users of diagnostic accuracy evidence, including researchers, clinicians, and policymakers, should evaluate studies of PHQ-9 accuracy with caution and ensure that recommendations regarding cutoff scores are based on adequately powered and analyzed primary studies or well-conducted meta-analyses.

## Notes

Supplement 1. **eMethods 1.** Details on the Methodology Used to Identify, Obtain, and Synthesize the Data Included in the Present Study **eMethods 2.** Search Strategies **eTable 1.** Characteristics of Included Primary Studies **eTable 2.** Frequencies of PHQ-9 Scores Among Participants With and Without Major Depression **eTable 3.** Differences in Accuracy Estimates of the Sample Optimal Cutoffs Clustered Into Categories Based on Distance From a Cutoff of ≥8 Compared to Accuracy Estimates From a Cutoff of ≥8 in the Population **eTable 4.** Variability of Data-Driven Optimal Cutoffs in 1,000 Samples of Size 100, 200, 500, and 1,000, Among Studies That Used the SCID as the Reference Standard **eTable 5.** Mean Bias of Accuracy Estimates (With 95% Confidence Intervals) for 1,000 Samples of Size 100, 200, 500, and 1,000, Among Studies That Used the SCID as the Reference Standard **eFigure.** Distribution of PHQ-9 Scores Among Individuals With and Without Major Depression

Supplement 2.
Data Sharing Statement
