# Supplementary material for: Data-Driven Cutoff Selection for the Patient Health Questionnaire-9 Depression Screening Tool
Source: JAMA Netw Open. 2024 Nov 22;7(11):e2429630. doi: 10.1001/jamanetworkopen.2024.29630 (PMC11584932; doi:10.1001/jamanetworkopen.2024.29630)
Supplement: Supplement 1. — eMethods 1. Details on the Methodology Used to Identify, Obtain, and Synthesize the Data Included in the Present Study eMethods 2. Search Strategies eTable 1. Characteristics of Included Primary Studies eTable 2. Frequencies of PHQ-9 Scores Among Participants With and Without Major Depression eTable 3. Differences in Accuracy Estimates of the Sample Optimal Cutoffs Clustered Into Categories Based on Distance From a Cutoff of ≥8 Compared to Accuracy Estimates From a Cutoff of ≥8 in the Population eTable 4. Variability of Data-Driven Optimal Cutoffs in 1,000 Samples of Size 100, 200, 500, and 1,000, Among Studies That Used the SCID as the Reference Standard eTable 5. Mean Bias of Accuracy Estimates (With 95% Confidence Intervals) for 1,000 Samples of Size 100, 200, 500, and 1,000, Among Studies That Used the SCID as the Reference Standard eFigure. Distribution of PHQ-9 Scores Among Individuals With and Without Major Depression [file jamanetwopen-e2429630-s001.pdf]

Supplemental Online Content

Levis B, Mani Bhandari PM, Neupane D, et al; for the DEPRESsion Screening Data (DEPRESSD) PHQ Group. Variability and bias in optimal cutoff selection and accuracy estimates of depression screening studies. *JAMA Netw Open*. 2024;7(11):e2429630. doi:10.1001/jamanetworkopen.2024.29630

eMethods 1. Details on the Methodology Used to Identify, Obtain, and Synthesize the Data Included in the Present Study

eMethods 2. Search Strategies

eTable 1. Characteristics of Included Primary Studies

eTable 2. Frequencies of PHQ-9 Scores Among Participants With and Without Major Depression

eTable 3. Differences in Accuracy Estimates of the Sample Optimal Cutoffs Clustered Into Categories Based on Distance From a Cutoff of ≥ 8 Compared to Accuracy Estimates From a Cutoff of ≥ 8 in the Population

eTable 4. Variability of Data-Driven Optimal Cutoffs in 1,000 Samples of Size 100, 200, 500, and 1,000, Among Studies That Used the SCID as the Reference Standard

eTable 5. Mean Bias of Accuracy Estimates (With 95% Confidence Intervals) for 1,000 Samples of Size 100, 200, 500, and 1,000, Among Studies That Used the SCID as the Reference Standard

eFigure. Distribution of PHQ-9 Scores Among Individuals With and Without Major Depression

This supplemental material has been provided by the authors to give readers additional information about their work.

eMethods1. Details on the methodology used to identify, obtain, and synthesize the data included in the present study

Study selection

In the main IPDMA, datasets from articles in any language were eligible for inclusion if (1) they included PHQ-9 scores; (2) they included diagnostic classifications for current Major Depressive Episode (MDE) or Major Depressive Disorder (MDD) based on DSM[A1-A3] or International Classification of Diseases[A4] criteria, using a validated semi-structured or fully structured interview; (3) the PHQ-9 and diagnostic interview were administered within two weeks of each other, since diagnostic criteria for major depression are for symptoms in the last two weeks; (4) participants were ≥ 18 years and not recruited from youth or school-based settings; and (5) participants were not recruited from psychiatric settings or because they were identified as having symptoms of depression, since screening is done to identify unrecognized cases. Datasets where not all participants were eligible were included if primary data allowed selection of eligible participants.

Data sources and searches

A medical librarian searched Medline, Medline In-Process & Other Non-Indexed Citations and PsycInfo via Ovid as well as Web of Science from January 1, 2000 to May 9, 2018, using a peer-reviewed[A5] search strategy (eMethods2). We also reviewed reference lists of relevant reviews and queried contributing authors about non-published studies. Search results were uploaded into RefWorks (RefWorks-COS, Bethesda, MD, USA). After de-duplication, unique citations were uploaded into DistillerSR (Evidence Partners, Ottawa, Canada) for processing review results.

Two investigators independently reviewed titles and abstracts for eligibility. If either investigator deemed a study potentially eligible, full-text review was done by two investigators,

independently, with disagreements resolved by consensus, consulting a third investigator when necessary. Translators were consulted for languages other than those for which team members were fluent.

Data contribution and synthesis

Authors of eligible datasets were invited to contribute de-identified primary data, including PHQ-9 scores and major depression status. We emailed corresponding authors of eligible primary studies at least three times, as necessary, with at least two weeks between each email. If we did not receive a response, we emailed co-authors and attempted to contact corresponding authors by phone.

Individual participant data were converted to a standard format and synthesized into a single dataset. We compared published participant characteristics and diagnostic accuracy results with results from raw datasets and resolved any discrepancies in consultation with the original investigators.

For defining major depression, we considered MDD or MDE based on the DSM or ICD. If more than one was reported, we prioritized MDE over MDD, since screening would attempt to detect depressive episodes and further interview would determine if the episode were related to MDD, bipolar disorder, or persistent depressive disorder, and DSM over ICD.

Search results and inclusion of primary datasets

Of 9,670 unique titles and abstracts identified from the database search, 9,199 were excluded after title and abstract review and 297 after full-text review, leaving 174 eligible articles with data from 118 unique participant samples, of which 91 (77%) contributed datasets. In addition, authors of included studies contributed data from nine unpublished studies, for a total of 100 datasets (N participants = 44,503 participants; N major depression = 4,541).

© 2024 Levis B et al. *JAMA Network Open*

eMethods2. Search Strategies

MEDLINE (OvidSP)

1. PHQ*.af.
2. patient health questionnaire*.af.
3. 1 or 2
4. Mass Screening/
5. Psychiatric Status Rating Scales/
6. "Predictive Value of Tests"/
7. "Reproducibility of Results"/
8. exp "Sensitivity and Specificity"/
9. Psychometrics/
10. Prevalence/
11. Reference Values/
12.. Reference Standards/
13. exp Diagnostic Errors/
14. Mental Disorders/di, pc [Diagnosis, Prevention & Control]
15. Mood Disorders/di, pc [Diagnosis, Prevention & Control]
16. Depressive Disorder/di, pc [Diagnosis, Prevention & Control]
17. Depressive Disorder, Major/di, pc [Diagnosis, Prevention & Control]
18. Depression, Postpartum/di, pc [Diagnosis, Prevention & Control]
19. Depression/di, pc [Diagnosis, Prevention & Control]
20. validation studies.pt.
21. comparative study.pt.
22. screen*.af.
23. prevalence.af.
24. predictive value*.af.
25. detect*.ti.
26. sensitiv*.ti.
27. valid*.ti.
28. revalid*.ti.
29. predict*.ti.
30. accura*.ti.
31. psychometric*.ti.
32. identif*.ti.
33. specificit*.ab.
34. cut?off*.ab.
35. cut* score*.ab.
36. cut?point*.ab.
37. threshold score*.ab.
38. reference standard*.ab.
39. reference test*.ab.
40. index test*.ab.
41. gold standard.ab.
42. or/4-41
43. 3 and 42
44. limit 43 to yr=”2000-Current”

PsycINFO (OvidSP)

1. PHQ*.af.
2. patient health questionnaire*.af.
3. 1 or 2

© 2024 Levis B et al. *JAMA Network Open*

4. Diagnosis/
5. Medical Diagnosis/
6. Psychodiagnosis/
7. Misdiagnosis/
8. Screening/
9. Health Screening/
10. Screening Tests/
11. Prediction/
12. Cutting Scores/
13. Psychometrics/
14. Test Validity/
15. screen*.af.
16. predictive value*.af.
17. detect*.ti.
18. sensitiv*.ti.
19. valid*.ti.
20. revalid*.ti.
21. accura*.ti.
22. psychometric*.ti.
23. specificit*.ab.
24. cut?off*.ab.
25. cut* score*.ab.
26. cut?point*.ab.
27. threshold score*.ab.
28. reference standard*.ab.
29. reference test*.ab.
30. index test*.ab.
31. gold standard.ab.
32. or/4-31
33. 3 and 32
34. Limit 33 to "2000 to current"

Web of Science (Web of Knowledge)

#1: TS= (PHQ* OR "Patient Health Questionnaire*")
#2: TS= (screen* OR prevalence OR "predictive value*" OR detect* OR sensitiv* OR valid* OR revalid* OR predict* OR accura* OR psychometric* OR identif* OR specificit* OR cutoff* OR "cut off*" OR "cut* score*" OR cutpoint* OR "cut point*" OR "threshold score*" OR "reference standard*" OR "reference test*" OR "index test*" OR "gold standard")
#1 AND #2
Indexes=SCI-EXPANDED, SSCI, A&HCI, CPCI-S, CPCI-SSH Timespan=2000-2018

© 2024 Levis B et al. *JAMA Network Open*

eTable1. Characteristics of included primary studies (N = 100)

First Author, Year	Country	Recruited Population	Diagnostic Interview	Classification System	Total N	Major Depression N (%)
**Semi-structured Interviews**						
Alamri, 2017[A6]	Saudi Arabia	Hospitalized elderly in medical and surgical wards	SCID	DSM-IV	199	24 (12)
Amoozegar, 2017[A7]	Canada	Migraine patients	SCID	DSM-IV	203	49 (24)
Amtmann, 2015[A8]	USA	Multiple sclerosis patients	SCID	DSM-IV	164	48 (29)
Ayalon, 2010[A9]	Israel	Elderly primary care patients	SCID	DSM-IV	151	6 (4)
Beraldi, 2014[A10]	Germany	Cancer inpatients	SCID	DSM-IV	116	7 (6)
Bernstein, 2018[A11]	Canada	IBD patients	SCID	DSM-IV	240	21 (9)
Bhana, 2015[A12]	South Africa	Chronic care patients	SCID	DSM-IV	679	78 (11)
Bombardier, 2012[A13]	USA	Inpatients with spinal cord injuries	SCID	DSM-IV	160	14 (9)
Chagas, 2013[A14]	Brazil	Outpatients with Parkinson's Disease	SCID	DSM-IV	84	19 (23)
Chibanda, 2016[A15]	Zimbabwe	A primary care population with high HIV prevalence	SCID	DSM-IV	264	149 (56)
Eack, 2006[A16]	USA	Women seeking psychiatric services for their children at two mental health centers	SCID	DSM-IV	48	12 (25)
Fann, 2005[A17]	USA	Inpatients with traumatic brain injury	SCID	DSM-IV	135	45 (33)
Fiest, 2014[A18]	Canada	Epilepsy outpatients	SCID	DSM-IV	169	23 (14)
Fischer, 2014[A19]	Germany	Heart failure patients	SCID	DSM-IV	194	11 (6)

© 2024 Levis B et al. *JAMA Network Open*

Gjerdingen, 2009[A20]	USA	Mothers registering their newborns for well-child visits at medical or pediatric clinics	SCID	DSM-IV	419	19 (5)
Gräfe, 2004[A21]	Germany	Medical and psychosomatic outpatients	SCID	DSM-IV	494	67 (14)
Green, 2017[A22]	USA	Returning veterans	SCID	DSM-V	176	22 (13)
Green, 2018[A23]	Kenya	Pregnant women and new mothers	SCID	DSM-V	192	10 (5)
Haroz, 2017[A24]	Myanmar	Primary care patients	SCID	DSM-IV	132	29 (22)
Hitchon, 2019[A25a]	Canada	Rheumatoid arthritis patients	SCID	DSM-IV	148	16 (11)
Khamseh, 2011[A26]	Iran	Type 2 diabetes patients	SCID	DSM-IV	184	79 (43)
Kwan, 2012[A27]	Singapore	Post-stroke inpatients undergoing rehabilitation	SCID	DSM-IV-TR	113	3 (3)
Lambert, 2015[A28]	Australia	Cancer patients	SCID	DSM-IV	147	21 (14)
Lara, 2015[A29]	Mexico	Pregnant women during the third trimester of pregnancy	SCID	DSM-IV	280	29 (10)
Liu, 2011[A30]	Taiwan	Primary care patients	SCAN	DSM-IV	1532	50 (3)
Marrie, 2018[A31]	Canada	Multiple sclerosis patients	SCID	DSM-IV	244	25 (10)
Martin-Subero, 2017[A32]	Spain	Medical inpatients	SCID	DSM-III	1003	83 (8)
McGuire, 2013[A33]	USA	Acute coronary syndrome inpatients	DISH	DSM-IV	100	9 (9)
Osório, 2009[A34]	Brazil	Women in primary care	SCID	DSM-IV	177	60 (34)
Osório, 2012[A35]	Brazil	Inpatients from various clinical wards	SCID	DSM-IV	86	28 (33)
Patten, 2015[A36]	Canada	Multiple sclerosis patients	SCID	DSM-IV	143	20 (14)
Picardi, 2005[A37]	Italy	Inpatients with skin diseases	SCID	DSM-IV	138	12 (9)

© 2024 Levis B et al. *JAMA Network Open*

Prisnie, 2016[A38]	Canada	Stroke and transient ischemic attack patients	SCID	DSM-IV	114	11 (10)
Quinn, Unpublished[A39a]	UK	Stroke patients	SCID	DSM-V	146	17 (12)
Richardson, 2010[A40]	USA	Older adults undergoing in-home aging services care management assessment	SCID	DSM-IV	377	95 (25)
Rooney, 2013[A41]	UK	Patients with cerebral glioma	SCID	DSM-IV	126	14 (11)
Shinn, 2017[A42]	USA	Cancer patients	SCID	DSM-IV	139	12 (9)
Sidebottom, 2012[A43]	USA	Pregnant women	SCID	DSM-IV	246	12 (5)
Simning, 2012[A44]	USA	Older adults living in public housing	SCID	DSM-IV	190	10 (5)
Spangenberg, 2015[A45]	Germany	Primary care patients	SCID	DSM-IV	160	1 (1)
Turner, 2012[A46]	Australia	Stroke patients	SCID	DSM-IV	72	13 (18)
Turner, Unpublished[A47a]	Australia	Cardiac rehabilitation patients	SCID	DSM-IV	51	4 (8)
Twist, 2013[A48]	UK	Type 2 diabetes outpatients	SCAN	DSM-IV	360	80 (22)
Vöhringer, 2013[A49]	Chile	Primary care patients	SCID	DSM-IV	190	59 (31)
Wagner, 2017[A50]	USA	Patients starting radiotherapy for the first diagnosis of any tumor	SCID	DSM-IV	54	6 (11)
Williams, 2012[A51]	USA	Parkinson’s Disease patients	SCID	DSM-IV	235	61 (26)
Wittkampf, 2009[A52]	The Netherlands	Primary care patients at risk for depression	SCID	DSM-IV	260	45 (17)
**Fully structured Interviews**						
Arroll, 2010[A53]	New Zealand	Primary care patients	CIDI	DSM-IV	2528	156 (6)
Azah, 2005[A54]	Malaysia	Adults attending family medicine clinics	CIDI	ICD-10	180	30 (17)

© 2024 Levis B et al. *JAMA Network Open*

de Man-van Ginkel, 2012[A55]	The Netherlands	Stroke patients	CIDI	DSM-IV	382	54 (14)
Delgadillo, 2011[A56]	UK	Injecting drug users	CIS-R	ICD-10	103	51 (50)
Fisher, 2016[A57]	Australia	Primiparous women less than 6 weeks postpartum	CIDI	DSM-IV	357	4 (1)
Gelaye, 2014[A58]	Ethiopia	Outpatients at a general hospital	CIDI	DSM-IV	923	162 (18)
Grool, 2011[A59]	The Netherlands	Non-demented patients with symptomatic atherosclerotic disease	CIDI	DSM-IV	477	22 (5)
Hahn, 2006[A60]	Germany	Patients with chronic illnesses from rehabilitation centers	CIDI	DSM-IV	211	18 (9)
Henkel, 2004[A61]	Germany	Primary care patients	CIDI	ICD-10	430	43 (10)
Hobfoll, 2011[A62]	Israel	Jewish and Palestinian residents of Jerusalem exposed to war	CIDI	DSM-IV	144	42 (29)
Kiely, 2014[A63]	Australia	Community sample of adults	CIDI	ICD-10	822	33 (4)
Kim, 2017[A64]	South Korea	Randomly selected adults	CIDI	DSM-IV	3071	205 (7)
Kohrt, 2016[A65]	Nepal	Primary care patients	CIDI	DSM-IV	125	17 (14)
Liu, 2015[A66]	Canada	Working population	CIDI	DSM-IV	4182	91 (2)
Mohd Sidik, 2012[A67]	Malaysia	Primary care patients	CIDI	DSM-IV	146	31 (21)
Patel, 2008[A68]	India	Primary care patients	CIS-R	ICD-10	299	13 (4)
Pence, 2012[A69]	Cameroon	HIV-infected patients	CIDI	DSM-IV	398	11 (3)
Razykov, 2013[A70]	Canada	Patients with systemic sclerosis	CIDI	DSM-IV	345	13 (4)
Thombs, 2008[A71]	USA	Outpatients with coronary artery disease	C-DIS	DSM-IV	1006	221 (22)

Zuithoff, 2009[A72]	The Netherlands	General practice patients	CIDI	DSM-IV	1038	135 (13)
**Mini International Neuropsychiatric Interviews (MINI)**						
Akena, 2013[A73]	Uganda	HIV/AIDS patients	MINI	DSM-IV	91	11 (12)
Baron, 2017[A74]	South Africa	Xhosa, Afrikaans and Zulu-speaking general population	MINI	DSM-IV	851	93 (11)
Buji, 2018[A75]	Malaysia	Patients with systemic lupus erythematosus	MINI	DSM-IV	130	5 (4)
Cholera, 2014[A76]	South Africa	Patients undergoing routine HIV counseling and testing at a primary health care clinic	MINI	DSM-IV	397	47 (12)
Conway, 2016[A77]	Australia	Heart transplant recipients	MINI	DSM-IV	26	2 (8)
de la Torre, 2016[A78]	Argentina	Hospitalized general medical patients	MINI	DSM-IV	257	69 (27)
Garabiles, 2020[A79a]	China	Female Filipino domestic workers in Macao	MINI	DSM-IV	99	39 (39)
Gholizadeh, 2019[A80a]	Iran	Coronary artery disease patients	MINI	DSM-IV	79	12 (15)
Hantsoo, 2017[A81]	USA	General population	MINI	DSM-IV	321	19 (6)
Hides, 2007[A82]	Australia	Injection drug users accessing a needle and syringe program	MINI	DSM-IV	103	47 (46)
Hyphantis, 2011[A83]	Greece	Patients with various rheumatologic disorders	MINI	DSM-IV	213	69 (32)
Hyphantis, 2014[A84]	Greece	Patients with chronic illnesses presenting at the emergency department	MINI	DSM-IV	349	95 (27)

© 2024 Levis B et al. *JAMA Network Open*

Inagaki, 2013[A85]	Japan	Internal medicine outpatients	MINI	DSM-III-R	104	21 (20)
Janssen, 2016[A86]	The Netherlands	General population and Type 2 diabetes patients	MINI	DSM-IV	4695	156 (3)
Lamers, 2008[A87]	The Netherlands	Elderly primary care patients with diabetes mellitus or chronic obstructive pulmonary disease	MINI	DSM-IV	104	59 (57)
Levin-Aspenson, 2017[A88]	USA	General population	MINI	DSM-V	408	66 (16)
Liu, 2016[A89]	China	General hospital outpatients	MINI	DSM-IV	1997	97 (5)
Lotrakul, 2008[A90]	Thailand	Outpatients	MINI	DSM-IV	278	19 (7)
Muramatsu, 2007[A91]	Japan	Primary care patients	MINI	DSM-IV	116	32 (28)
Muramatsu, 2018[A92]	Japan	Primary care patients	MINI	DSM-IV	152	46 (30)
Nakku, 2016[A93]	Uganda	Primary patients and hospital outpatients	MINI	DSM-IV	153	84 (55)
Paika, 2017[A94]	Greece	Patients with long term medical conditions	MINI	DSM-IV	474	98 (21)
Persoons, 2001[A95]	Belgium	Inpatients and patients at gastroenterological and hepatology wards	MINI	DSM-IV	173	28 (16)
Rancans, 2018[A96]	Latvia	Primary care patients	MINI	DSM-IV	1467	147 (10)
Santos, 2013[A97]	Brazil	General population	MINI	DSM-IV	196	25 (13)
Stafford, 2007[A98]	Australia	Inpatients with coronary artery disease who had undergone surgery	MINI	DSM-IV	193	35 (18)
Sung, 2013[A99]	Singapore	Primary care patients	MINI	DSM-IV	399	12 (3)
Suzuki, 2015[A100]	Japan	Outpatients in general medicine department	MINI	DSM-IV	511	42 (8)
van Heyningen, 2018[A101]	South Africa	Pregnant women	MINI	DSM-IV	373	81 (22)

© 2024 Levis B et al. *JAMA Network Open*

van Steenbergen-Weijenburg, 2010[A102]	The Netherlands	Diabetes patients	MINI	DSM-IV	196	37 (19)
Volker, 2016[A103]	The Netherlands	Employees on sickness leave	MINI	DSM-IV	93	23 (25)
Wang, 2014[A104]	China	General population	MINI	DSM-IV	1036	28 (3)
Zhang, 2013[A105]	Hong Kong, China	Type 2 diabetes patients	MINI	DSM-IV	68	17 (25)

Abbreviations: C-DIS: Computerized Diagnostic Interview Schedule; CIDI: Composite International Diagnostic Interview; CIS-R: Clinical Interview Schedule Revised; DISH: Depression Interview and Structured Hamilton; DSM: Diagnostic and Statistical Manual of Mental Disorders; ICD: International Classification of Diseases; MINI: Mini Neuropsychiatric Diagnostic Interview; SCAN: Schedules for Clinical Assessment in Neuropsychiatry; SCID: Structured Clinical Interview for DSM Disorders; UK: United Kingdom; USA: United States of America.

[a]Was unpublished at the time of electronic database search

eTable2. Frequencies of PHQ-9 scores among participants with and without major depression

PHQ-9 score	N participants with PHQ-9 score	N (%) with major depression among those with PHQ-9 score	N (%) without major depression among those with PHQ-9 score
**0**	8,811	89 (1%)	8722 (99%)
**1**	5,109	51 (1%)	5058 (99%)
**2**	4,795	63 (1%)	4732 (99%)
**3**	4,195	81 (2%)	4114 (98%)
**4**	3,463	103 (3%)	3360 (97%)
**5**	2,880	141 (5%)	2739 (95%)
**6**	2,379	182 (8%)	2197 (92%)
**7**	2,012	181 (9%)	1831 (91%)
**8**	1,744	213 (12%)	1531 (88%)
**9**	1,501	221 (15%)	1280 (85%)
**10**	1,136	235 (21%)	901 (79%)
**11**	951	239 (25%)	712 (75%)
**12**	921	286 (31%)	635 (69%)
**13**	740	259 (35%)	481 (65%)
**14**	698	275 (39%)	423 (61%)
**15**	573	266 (46%)	307 (54%)
**16**	508	277 (55%)	231 (45%)
**17**	398	216 (54%)	182 (46%)
**18**	367	198 (54%)	169 (46%)
**19**	265	166 (63%)	99 (37%)
**20**	231	161 (70%)	70 (30%)
**21**	217	156 (72%)	61 (28%)
**22**	149	112 (75%)	37 (25%)
**23**	131	96 (73%)	35 (27%)
**24**	120	95 (79%)	25 (21%)
**25**	93	76 (82%)	17 (18%)
**26**	34	31 (91%)	3 (9%)
**27**	82	72 (88%)	10 (12%)
**Total**	44,503	4,541 (10%)	39,962 (90%)

© 2024 Levis B et al. *JAMA Network Open*

eTable3. Bias[a] in accuracy estimates of the sample optimal cutoffs clustered into categories based on distance from a cutoff of ≥ 8 compared to accuracy estimates from a cutoff of ≥ 8 in the population

Optimal cutoff	Sample size = 100			Sample size = 200			Sample size = 500			Sample size = 1,000		
	N	Sensitivity	Specificity	N	Sensitivity	Specificity	N	Sensitivity	Specificity	N	Sensitivity	Specificity
**≤ 5**	93	15.2 (13.8, 16.5)	-17.2 (-18.5, -15.8)	69	14 (12.8, 15.3)	-16.3 (-17.5, -15.2)	30	11.8 (10.2, 13.4)	-15.9 (-16.8, -15)	6	12.3 (10.8, 13.8)	-15.8 (-17.2, -14.4)
**6 – 7**	279	10 (8.9, 11.0)	-6 (-6.6, -5.4)	319	9.7 (9, 10.4)	-6.1 (-6.6, -5.7)	319	6.8 (6.2, 7.3)	-5.9 (-6.3, -5.6)	353	6.2 (5.8, 6.6)	-5.9 (-6.2, -5.6)
**8**	165	7.4 (5.9, 8.9)	0.5 (-0.1, 1.0)	215	5.6 (4.7, 6.6)	0.1 (-0.3, 0.4)	264	2.5 (1.9, 3.1)	0.2 (0.0, 0.5)	333	1.4 (1.0, 1.8)	0.1 (0.0, 0.3)
**9 – 10**	250	4.4 (3.2, 5.7)	5.5 (5.0, 6.0)	276	0.7 (-0.3, 1.7)	5.5 (5.1, 5.8)	333	-1.5 (-2.1, -1.0)	5.2 (4.9, 5.4)	295	-2.9 (-3.3, -2.4)	4.7 (4.5, 4.9)
**≥ 11**	213	-0.5 (-2.2, 1.2)	11.3 (10.9, 11.7)	121	-4.6 (-6.3, -2.9)	10.5 (10.1, 11)	54	-8.2 (-9.5, -6.9)	10 (9.6, 10.4)	13	-7.3 (-9.0, -5.5)	9.4 (8.7, 10.0)

N = Number of samples, NA = Not applicable
Differences are presented as mean difference (95% confidence interval)
[a] Calculated as sample-specific optimal cutoff – population optimal cutoff of ≥ 8, where sample-specific optimal cutoff refers to the cutoff maximizing Youden's J in each simulated sample, and the optimal cutoff in the full PHQ-9 IPDMA dataset is ≥ 8 (sensitivity = 80.4%, specificity = 82.0%)

eTable4. Variability of data-driven optimal cutoffs in 1,000 samples of size 100, 200, 500, and 1,000, among studies that used the SCID as the reference standard[a]

Sample-specific optimal cutoff	Sample size = 100 N samples	Sample size = 200 N samples	Sample size = 500 N samples	Sample size = 1,000 N samples
**≥ 5**	7			
**≥ 6**	17	5		
**≥ 7**	47	35	11	1
**≥ 8**	79	67	32	8
**≥ 9**	135	145	134	94
**≥ 10**	150	193	230	252
**≥ 11**	210	294	425	546
**≥ 12**	192	176	150	95
**≥ 13**	70	44	16	4
**≥ 14**	63	32	2	
**≥ 15**	20	9		
**≥ 16**	5			
**≥ 17**	3			
**≥ 18**	2			

SCID, Structured Clinical Interview for DSM

[a] N studies = 44, N participants = 9242, N major depression = 1389

© 2024 Levis B et al. *JAMA Network Open*

eTable5. Mean bias of accuracy estimates (with 95% confidence intervals) for 1,000 samples of size 100, 200, 500, and 1,000, among studies that used the SCID as the reference standard[a]

	**Sample size = 100**		**Sample size = 200**		**Sample size = 500**		**Sample size = 1,000**	
	**Sensitivity**	**Specificity**	**Sensitivity**	**Specificity**	**Sensitivity**	**Specificity**	**Sensitivity**	**Specificity**
**Sample-specific optimal cutoff[b] – Population optimal cutoff of ≥ 11[c]**	6.8 (6.2, 7.4)	-1.9 (-2.4, -1.4)	4.4 (3.9, 4.9)	-1.7 (-2.1, -1.3)	2.6 (2.3, 2.9)	-1.7 (-1.4, 2.0)	2.0 (1.8, 2.2)	-1.5 (-1.7, -1.3)

Differences are presented as mean difference (95% confidence interval)

SCID, Structured Clinical Interview for DSM

[a] N studies = 44, N participants = 9242, N major depression = 1389

[b] Sample-specific optimal cutoff refers to the cutoff maximizing Youden's J in each simulated sample

[c] the optimal cutoff in the subset of the PHQ-9 IPDMA dataset that used the SCID as the reference standard is ≥ 11 (sensitivity = 78.1%, specificity = 85.3%)

© 2024 Levis B et al. *JAMA Network Open*

eFigure 1. Distribution of PHQ-9 scores among individuals with and without major depression

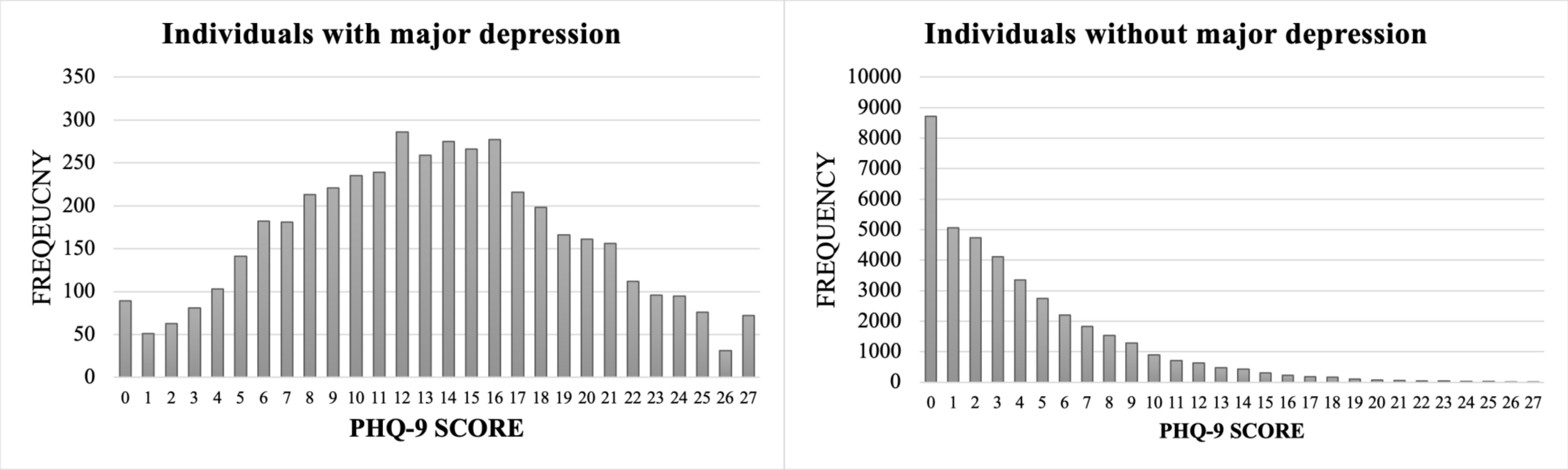

© 2024 Levis B et al. *JAMA Network Open*

REFERENCES

A1. Diagnostic and statistical manual of mental disorders: DSM-III 3rd ed, revised. Washington, DC: American Psychiatric Association 1987.

A2. Diagnostic and statistical manual of mental disorders: DSM-IV 4th ed. Washington, DC: American Psychiatric Association 1994.

A3. Diagnostic and statistical manual of mental disorders: DSM-IV 4th ed, text revised. Washington, DC: American Psychiatric Association 2000.

A4. The ICD-10 Classifications of Mental and Behavioural Disorder: Clinical Descriptions and Diagnostic Guidelines Geneva: World Health Organization 1992.

A5. Sampson M, McGowan J, Lefebvre C, Moher D, Grimshaw J. PRESS: peer review of electronic search strategies. *Ottawa: Canadian Agency for Drugs and Technologies in Health*. 2008.

A6. Alamri SH, Bari AI, Ali AT. Depression and associated factors in hospitalized elderly: a cross-sectional study in a Saudi teaching hospital. *Ann Saudi Med* 2017;37:122-129. doi:10.5144/0256-4947.2017.122

A7. Amoozegar F, Patten SB, Becker WJ, et al. The prevalence of depression and the accuracy of depression screening tools in migraine patients. *Gen Hosp Psychiatry* 2017;48:25-31. doi: 10.1016/j.genhosppsych.2017.06.006

A8. Amtmann D, Bamer AM, Johnson KL, et al. A comparison of multiple patient reported outcome measures in identifying major depressive disorder in people with multiple sclerosis. *J Psychosom Res* 2015;79:550-557. doi:10.1016/j.jpsychores.2015.08.007

A9. Ayalon L, Goldfracht M, Bech P. 'Do you think you suffer from depression?' Re-evaluating the use of a single item question for the screening of depression in older primary care patients. *Int J Geriatr Psychiatry* 2010;25:497-502. doi: 10.1002/gps.2368

A10. Beraldi A, Baklayan A, Hoster E, et al. Which questionnaire is most suitable for the detection of depressive disorders in haemato-oncological patients? Comparison between HADS, CES-D and PHQ-9. *Oncol Res Treat* 2014;37:108–109.

A11. Bernstein CN, Zhang L, Lix LM, et al. The validity and reliability of screening measures for depression and anxiety disorders in inflammatory bowel disease. *Inflamm Bowel Dis* 2018;24:1867-1875. doi: 10.1093/ibd/izy068

A12. Bhana A, Rathod SD, Selohilwe O, et al. The validity of the Patient Health Questionnaire for screening depression in chronic care patients in primary health care in South Africa. *BMC psychiatry* 2015;15:118. doi: 10.1186/s12888-015-0503-0

A13. Bombardier CH, Kalpakjian CZ, Graves DE, et al. Validity of the Patient Health Questionnaire-9 in assessing major depressive disorder during inpatient spinal cord injury rehabilitation. *Arch Phys Med Rehabil* 2012;93:1838-1845. doi: 10.1016/j.apmr.2012.04.019

A14. Chagas MH, Tumas V, Rodrigues GR, et al. Validation and internal consistency of Patient Health Questionnaire-9 for major depression in Parkinson's disease. *Age Ageing* 2013;42:645-49. doi: 10.1093/ageing/aft065

A15. Chibanda D, Verhey R, Gibson LJ, et al. Validation of screening tools for depression and anxiety disorders in a primary care population with high HIV prevalence in Zimbabwe. *J Affect Disord* 2016;198:50-55. doi: 10.1016/j.jad.2016.03.006

A16. Eack SM, Greeno CG, Lee BJ. Limitations of the Patient Health Questionnaire in identifying anxiety and depression in community mental health: many cases are undetected. *Res Soc Work Pract* 2006;16:625-631. doi: 10.1177/1049731506291582

A17. Fann JR, Bombardier CH, Dikmen S, et al. Validity of the Patient Health Questionnaire-9 in assessing depression following traumatic brain injury. *J Head Trauma Rehabil* 2005;20:501-511. doi: 10.1097/00001199-200511000-00003

A18. Fiest KM, Patten SB, Wiebe S, et al. Validating screening tools for depression in epilepsy. *Epilepsia* 2014;55:1642-1650. doi: 10.1111/epi.12754

A19. Fischer HF, Klug C, Roeper K, et al. Screening for mental disorders in heart failure patients using computer-adaptive tests. *Qual Life Res* 2014;23:1609-1618. doi: 10.1007/s11136-013-0599-y

A20. Gjerdingen D, Crow S, McGovern P, et al. Postpartum depression screening at well-child visits: validity of a 2-question screen and the PHQ-9. *Ann Fam Med* 2009;7:63-70. doi: 10.1370/afm.933

A21. Gräfe K, Zipfel S, Herzog W, et al. Screening for psychiatric disorders with the Patient Health Questionnaire (PHQ). Results from the German validation study. *Diagnostica* 2004;50:171-181. doi: 10.1026/0012-1924.50.4.171

A22. Green JD, Annunziata A, Kleiman SE, et al. Examining the diagnostic utility of the DSM-5 PTSD symptoms among male and female returning veterans. *Depress Anxiety* 2017;34:752-760. doi: 10.1002/da.22667

A23. Green EP, Tuli H, Kwobah E, et al. Developing and validating a perinatal depression screening tool in Kenya blending Western criteria with local idioms: a mixed methods study. *J Affect Disord* 2018;228:49-59. doi: 10.1016/j.jad.2017.11.027

A24. Haroz EE, Bass J, Lee C, et al. Development and cross-cultural testing of the International Depression Symptom Scale (IDSS): a measurement instrument designed to represent global presentations of depression. *Glob Ment Health (Camb)* 2017;4:e17. doi: 10.1017/gmh.2017.16

A25. Hitchon CA, Zhang L, Peschken CA, et al. Validity and reliability of screening measures for depression and anxiety disorders in rheumatoid arthritis. *Arthritis Care Res (Hoboken)* 2020;72:1130-1139. doi: 10.1002/acr.24011

© 2024 Levis B et al. *JAMA Network Open*

A26. Khamseh ME, Baradaran HR, Javanbakht A, et al. Comparison of the CES-D and PHQ-9 depression scales in people with type 2 diabetes in Tehran, Iran. *BMC Psychiatry* 2011;11:61. doi: 10.1186/1471-244X-11-61

A27. Kwan Y, Tham WY, Ang A. Validity of the Patient Health Questionnaire-9 (PHQ-9) in the screening of post-stroke depression in a multi-ethnic population. *Biol Psychiatry* 2012;71:141S-141S. doi: 10.1016/j.biopsych.2012.02.013

A28. Lambert SD, Clover K, Pallant JF, et al. Making sense of variations in prevalence estimates of depression in cancer: a co-calibration of commonly used depression scales using Rasch analysis. *J Natl Compr Canc Netw* 2015;13:1203-1211. doi: 10.6004/jnccn.2015.0149

A29. Lara MA, Navarrete L, Nieto L, et al. Prevalence and incidence of perinatal depression and depressive symptoms among Mexican women. *J Affect Disord* 2015;175:18-24. doi: 10.1016/j.jad.2014.12.035

A30. Liu SI, Yeh ZT, Huang HC, et al. Validation of Patient Health Questionnaire for depression screening among primary care patients in Taiwan. *Compr Psychiatry* 2011;52:96-101. doi: 10.1016/j.comppsych.2010.04.013

A31. Marrie RA, Zhang L, Lix LM, et al. The validity and reliability of screening measures for depression and anxiety disorders in multiple sclerosis. *Mult Scler Relat Disord* 2018;20:9-15. doi: 10.1016/j.msard.2017.12.007

A32. Martin-Subero M, Kroenke K, Diez-Quevedo C, et al. Depression as measured by PHQ-9 versus clinical diagnosis as an independent predictor of long-term mortality in a prospective cohort of medical inpatients. *Psychosom Med* 2017;79:273-282. doi: 10.1097/PSY.0000000000000390

© 2024 Levis B et al. *JAMA Network Open*

A33. McGuire AW, Eastwood JA, Macabasco-O'Connell A, et al. Depression screening: utility of the Patient Health Questionnaire in patients with acute coronary syndrome. *Am J Crit Care* 2013;22:12-19. doi: 10.4037/ajcc2013899

A34. Osório FL, Vilela Mendes A, Crippa JA, et al. Study of the discriminative validity of the PHQ-9 and PHQ-2 in a sample of Brazilian women in the context of primary health care. *Perspect Psychiatr Care* 2009;45:216-227. doi: 10.1111/j.1744-6163.2009.00224.x

A35. Osório FL, Carvalho AC, Fracalossi TA, et al. Are two items sufficient to screen for depression within the hospital context? *Int J Psychiatry Med* 2012;44:141-148. doi: 10.2190/PM.44.2.e

A36. Patten SB, Burton JM, Fiest KM, et al. Validity of four screening scales for major depression in MS. *Mult Scler* 2015;21:1064-1071. doi: 10.1177/1352458514559297

A37. Picardi A, Adler DA, Abeni D, et al. Screening for depressive disorders in patients with skin diseases: a comparison of three screeners. *Acta Derm Venereol* 2005;85:414-419. doi: 10.1080/00015550510034966

A38. Prisnie JC, Fiest KM, Coutts SB, et al. Validating screening tools for depression in stroke and transient ischemic attack patients. *Int J Psychiatry Med* 2016;51:262-277. doi: 10.1177/0091217416652616

A39. Quinn TJ, Taylor-Rowan M, Elliott E, et al. Research protocol - Assessing Post-Stroke Psychology Longitudinal Evaluation (APPLE) study: A prospective cohort study in stroke. *Cereb Circ Cogn Behav* 2022;3:100042. doi: 10.1016/j.cccb.2022.100042.

A40. Richardson TM, He H, Podgorski C, et al. Screening depression aging services clients. *Am J Geriatr Psychiatry* 2010;18:1116-1123. doi: 10.1097/JGP.0b013e3181dd1c26

© 2024 Levis B et al. *JAMA Network Open*

A41. Rooney AG, McNamara S, Mackinnon M, et al. Screening for major depressive disorder in adults with cerebral glioma: an initial validation of 3 self-report instruments. *Neuro Oncol* 2013;15:122-129. doi: 10.1093/neuonc/nos282

A42. Shinn EH, Valentine A, Baum G, et al. Comparison of four brief depression screening instruments in ovarian cancer patients: diagnostic accuracy using traditional versus alternative cutpoints. *Gynecol Oncol* 2017;145:562-568. doi: 10.1016/j.ygyno.2017.03.024

A43. Sidebottom AC, Harrison PA, Godecker A, et al. Validation of the Patient Health Questionnaire (PHQ)-9 for prenatal depression screening. *Arch Womens Ment Health* 2012;15:367-374. doi: 10.1007/s00737-012-0295-x

A44. Simning A, van Wijngaarden E, Fisher SG, et al. Mental healthcare need and service utilization in older adults living in public housing. *Am J Geriatr Psychiatry* 2012;20:441-451. doi: 10.1097/JGP.0b013e31822003a7

A45. Spangenberg L, Glaesmer H, Boecker M, et al. Differences in Patient Health Questionnaire and Aachen Depression Item Bank scores between tablet versus paper-and-pencil administration. *Qual Life Res* 2015;24:3023-3032. doi: 10.1007/s11136-015-1040-5

A46. Turner A, Hambridge J, White J, et al. Depression screening in stroke: a comparison of alternative measures with the structured diagnostic interview for the Diagnostic and Statistical Manual of Mental Disorders, Fourth Edition (major depressive episode) as criterion standard. *Stroke* 2012;43:1000-1005. doi: 10.1161/STROKEAHA.111.643296

A47. Turner, A, Clover, K, Carter, G, Hambridge, J, White, J, Nelson, L, Alston, M, & Baker, A. Touchscreen: A new approach to depression screening in CVD patients. Heart Foundation Conference 2011: Heart to Heart: from Access to Action, March 17 - 19, 2011. * Winner 2nd Poster Prize

A48. Twist K, Stahl D, Amiel SA, et al. Comparison of depressive symptoms in type 2 diabetes using a two-stage survey design. *Psychosom Med* 2013;75:791-797. doi: 10.1097/PSY.0b013e3182a2b108

A49. Vöhringer PA, Jimenez MI, Igor MA, et al. Detecting mood disorder in resource-limited primary care settings: comparison of a self-administered screening tool to general practitioner assessment. *J Med Screen* 2013;20:118-124. doi: 10.1177/0969141313503954

A50. Wagner LI, Pugh SL, Small Jr W, et al. Screening for depression in cancer patients receiving radiotherapy: feasibility and identification of effective tools in the NRG Oncology RTOG 0841 trial. *Cancer* 2017;123:485-93. doi: 10.1002/cncr.29969

A51. Williams JR, Hirsch ES, Anderson K, et al. A comparison of nine scales to detect depression in Parkinson disease: which scale to use? *Neurology* 2012;78:998-1006. doi: 10.1212/WNL.0b013e31824d587f

A52. Wittkampf K, van Ravesteijn H, Baas K, et al. The accuracy of Patient Health Questionnaire-9 in detecting depression and measuring depression severity in high-risk groups in primary care. *Gen Hosp Psychiatry* 2009;31:451-459. doi: 10.1016/j.genhosppsych.2009.06.001

A53. Arroll B, Goodyear-Smith F, Crengle S, et al. Validation of PHQ-2 and PHQ-9 to screen for major depression in the primary care population. *Ann Fam Med* 2010;8:348-53. doi: 10.1370/afm.1139

A54. Azah MN, Shah ME, Juwita S, et al. Validation of the Malay version brief Patient Health Questionnaire (PHQ-9) among adult attending family medicine clinics. *International Medical Journal* 2005;12:259-63.

A55. De Man-van Ginkel JM, Hafsteinsdóttir T, Lindeman E, et al. An efficient way to detect poststroke depression by subsequent administration of a 9-item and a 2-item Patient Health Questionnaire. *Stroke* 2012;43:854-56. doi: 10.1161/STROKEAHA.111.640276

© 2024 Levis B et al. *JAMA Network Open*

A56. Delgadillo J, Payne S, Gilbody S, et al. How reliable is depression screening in alcohol and drug users? A validation of brief and ultra-brief questionnaires. *J Affect Disord* 2011;134:266-71. doi: 10.1016/j.jad.2011.06.017

A57. Fisher J, Rowe H, Wynter K, et al. Gender-informed, psychoeducational programme for couples to prevent postnatal common mental disorders among primiparous women: cluster randomised controlled trial. *BMJ Open* 2016;6:e009396. doi:10.1136/bmjopen-2015- 009396

A58. Gelaye B, Tadesse MG, Williams MA, et al. Assessing validity of a depression screening instrument in the absence of a gold standard. *Ann Epidemiol* 2014;24:527-31. doi: 10.1016/j.annepidem.2014.04.009

A59. Grool AM, van der Graaf Y, Mali WP, et al. Location of cerebrovascular and degenerative changes, depressive symptoms and cognitive functioning in later life: the SMART-Medea study. *J Neurol Neurosurg Psychiatry* 2011;82:1093-1100. doi:10.1136/jnnp.2010.232413

A60. Hahn D, Reuter K, Harter M. Screening for affective and anxiety disorders in medical patients - comparison of HADS, GHQ-12 and Brief-PHQ. *Psychsoc Med* 2006;3.

A61. Henkel V, Mergl R, Kohnen R, et al. Use of brief depression screening tools in primary care: consideration of heterogeneity in performance in different patient groups. *Gen Hosp Psychiatry* 2004;26:190-98. doi: 10.1016/j.genhosppsych.2004.02.003

A62. Hobfoll SE, Canetti D, Hall BJ, et al. Are community studies of psychological trauma's impact accurate? A study among Jews and Palestinians. *Psychol Assess* 2011;23:599-605. doi: 10.1037/a0022817

A63. Kiely KM, Butterworth P. Validation of four measures of mental health against depression and generalized anxiety in a community based sample. *Psychiatry Res* 2015;225:291-98. doi: 10.1016/j.psychres.2014.12.023

© 2024 Levis B et al. *JAMA Network Open*

A64. Kim DJ, Kim K, Lee HW, et al. Internet game addiction, depression, and escape from negative emotions in adulthood: a nationwide community sample of Korea. *J Nerv Ment Dis* 2017;205:568-73. doi: 10.1097/NMD.0000000000000698

A65. Kohrt BA, Luitel NP, Acharya P, et al. Detection of depression in low resource settings: validation of the Patient Health Questionnaire (PHQ-9) and cultural concepts of distress in Nepal. *BMC Psychiatry* 2016;16:58. doi: 10.1186/s12888-016-0768-y

A66. Liu Y, Wang J. Validity of the Patient Health Questionnaire-9 for DSM-IV major depressive disorder in a sample of Canadian working population. *J Affect Disord* 2015;187:122-26. doi: 10.1016/j.jad.2015.07.044

A67. Mohd Sidik S, Arroll B, Goodyear-Smith F. Criterion validity of the PHQ-9 (Malay version) in a primary care clinic in Malaysia. *Med J Malaysia* 2012;67:309-15.

A68. Patel V, Araya R, Chowdhary N, et al. Detecting common mental disorders in primary care in India: a comparison of five screening questionnaires. *Psychol Med* 2008;38:221-28. doi: 10.1017/S0033291707002334

A69. Pence BW, Gaynes BN, Atashili J, et al. Validity of an interviewer-administered Patient Health Questionnaire-9 to screen for depression in HIV-infected patients in Cameroon. *J Affect Disord* 2012;143:208-13. doi: 10.1016/j.jad.2012.05.056

A70. Razykov I, Hudson M, Baron M, et al. Utility of the Patient Health Questionnaire-9 to assess suicide risk in patients with systemic sclerosis. *Arthritis Care Res (Hoboken)* 2013;65:753-58. doi: 10.1002/acr.21894

A71. Thombs BD, Ziegelstein RC, Whooley MA. Optimizing detection of major depression among patients with coronary artery disease using the Patient Health Questionnaire: data from the heart and soul study. *J Gen Intern Med* 2008;23:2014-17. doi: 10.1007/s11606-008-0802-y

© 2024 Levis B et al. *JAMA Network Open*

A72. Zuithoff NP, Vergouwe Y, King M, et al. A clinical prediction rule for detecting major depressive disorder in primary care: the PREDICT-NL study. *Fam Pract* 2009;26:241-50. doi: 10.1093/fampra/cmp036

A73. Akena D, Joska J, Obuku EA, et al. Sensitivity and specificity of clinician administered screening instruments in detecting depression among HIV-positive individuals in Uganda. *AIDS Care* 2013;25:1245-52. doi: 10.1080/09540121.2013.764385

A74. Baron EC, Davies T, Lund C. Validation of the 10-item Centre for Epidemiological Studies Depression scale (CES-D-10) in Zulu, Xhosa and Afrikaans populations in South Africa. *BMC Psychiatry* 2017;17:6. doi: 10.1186/s12888-016-1178-x

A75. Buji RI, Abdul Murad NA, Chan LF, et al. Suicidal ideation in systemic lupus erythematosus: NR2A gene polymorphism, clinical and psychosocial factors. *Lupus* 2018;27:744-52. doi: 10.1177/0961203317742711

A76. Cholera R, Gaynes BN, Pence BW, et al. Validity of the Patient Health Questionnaire-9 to screen for depression in a high-HIV burden primary healthcare clinic in Johannesburg, South Africa. *J Affect Disord* 2014;167:160-66. doi: 10.1016/j.jad.2014.06.003

A77. Conway A, Sheridan J, Maddicks-Law J, et al. Accuracy of anxiety and depression screening tools in heart transplant recipients. *Appl Nurs Res* 2016;32:177-81. doi: 10.1016/j.apnr.2016.07.015

A78. de la Torre AY, Oliva N, Echevarrieta PL, et al. Major depression in hospitalized Argentine general medical patients: prevalence and risk factors. *J Affect Disord* 2016;197:36-42. doi: 10.1016/j.jad.2016.02.066

A79. Garabiles MR, Lao CK, Yip P, Chan EWW, Mordeno I, Hall BJ. Psychometric validation of PHQ-9 and GAD-7 in Filipino migrant domestic workers in Macao (SAR), China. *J Pers Assess* 2020;102(6):833-844. doi: 10.1080/00223891.2019.1644343.

© 2024 Levis B et al. *JAMA Network Open*

A80. Gholizadeh L, Shahmansouri N, Heydari M, et al. Assessment and detection of depression in patients with coronary artery disease: validation of the Persian version of the PHQ-9. *Contemp Nurse* 2019;55:185-94. doi: 10.1080/10376178.2019.1641119

A81. Hantsoo L, Podcasy J, Sammel M, et al. Pregnancy and the acceptability of computer-based versus traditional mental health treatments. *J Womens Health (Larchmt)* 2017;26:1106-13. doi: 10.1089/jwh.2016.6255

A82. Hides L, Lubman DI, Devlin H, et al. Reliability and validity of the Kessler 10 and Patient Health Questionnaire among injecting drug users. Aust N Z Psychiatry 2007;41:166-68. doi: 10.1080/00048670601109949

A83. Hyphantis T, Kotsis K, Voulgari PV, et al. Diagnostic accuracy, internal consistency, and convergent validity of the Greek version of the Patient Health Questionnaire 9 in diagnosing depression in rheumatologic disorders. *Arthritis Care Res (Hoboken)* 2011;63:1313-21. doi: 10.1002/acr.20505.

A84. Hyphantis T, Kroenke K, Papatheodorou E, et al. Validity of the Greek version of the PHQ 15-item Somatic Symptom Severity Scale in patients with chronic medical conditions and correlations with emergency department use and illness perceptions. *Compr Psychiatry* 2014;55:1950-59. doi: 10.1016/j.comppsych.2014.08.042

A85. Inagaki M, Ohtsuki T, Yonemoto N, et al. Validity of the Patient Health Questionnaire (PHQ)-9 and PHQ-2 in general internal medicine primary care at a Japanese rural hospital: a cross-sectional study. *Gen Hosp Psychiatry* 2013;35:592-97. doi: 10.1016/j.genhosppsych.2013.08.001

A86. Janssen EP, Köhler S, Stehouwer CD, et al. The Patient Health Questionnaire-9 as a screening tool for depression in individuals with type 2 diabetes mellitus: the Maastricht study. *J Am Geriatr Soc* 2016;64:e201-06. doi: 10.1111/jgs.14388

A87. Lamers F, Jonkers CC, Bosma H, et al. Summed score of the Patient Health Questionnaire-9 was a reliable and valid method for depression screening in chronically ill elderly patients. *J Clin Epidemiol* 2008;61:679-87. doi: 10.1016/j.jclinepi.2007.07.018

A88. Levin-Aspenson HF, Watson D. Mode of administration effects in psychopathology assessment: analyses of sex, age, and education differences in self-rated versus interview-based depression. *Psychol Assess* 2018;30:287-295. doi: 10.1037/pas0000474

A89. Liu h, He YL, Miao JM, et al. Validity and reliability of the 12-item Short Form Health Survey in outpatients from traditional Chinese internal department. *Chinese Mental Health Journal* 2016;30:6-12.

A90. Lotrakul M, Sumrithe S, Saipanish R. Reliability and validity of the Thai version of the PHQ-9. *BMC Psychiatry* 2008;8:46. doi: 10.1186/1471-244X-8-46

A91. Muramatsu K, Miyaoka H, Kamijima K, et al. The Patient Health Questionnaire, Japanese version: validity according to the Mini-International Neuropsychiatric Interview-Plus. *Psychol Rep* 2007;101:952-60. doi: 10.2466/pr0.101.3.952-960.

A92. Muramatsu K, Miyaoka H, Kamijima K, et al. Performance of the Japanese version of the Patient Health Questionnaire-9 (J-PHQ-9) for depression in primary care. *Gen Hosp Psychiatry* 2018;52:64-9. doi: 10.1016/j.genhosppsych.2018.03.007

A93. Nakku JE, Rathod SD, Kizza D, et al. Validity and diagnostic accuracy of the Luganda version of the 9-item and 2-item Patient Health Questionnaire for detecting major depressive disorder in rural Uganda. *Glob Ment Health (Camb)* 2016;3:e20. doi: 10.1017/gmh.2016.14

A94. Paika V, Andreoulakis E, Ntountoulaki E, et al. The Greek-Orthodox version of the Brief Religious Coping (B-RCOPE) instrument: psychometric properties in three samples and associations with mental disorders, suicidality, illness perceptions, and quality of life. *Ann Gen Psychiatry* 2017;16:13. doi: 10.1186/s12991-017-0136-4

A95. Persoons P, Luyckx K, Fischler B. Psychiatric diagnoses in Gastroenterolgy: validation of a self-report instrument (PRIME-MD Patient Health Questionnaire), epidemiology and recognition. *Gastroenterology* 2001;120:A114-A114. doi: 10.1016/S0016-5085(08)80560-4

A96. Rancans E, Trapencieris M, Ivanovs R, et al. Validity of the PHQ-9 and PHQ-2 to screen for depression in nationwide primary care population in Latvia. *Ann Gen Psychiatry* 2018;17:33. doi: 10.1186/s12991-018-0203-5

A97. Santos IS, Tavares BF, Munhoz TN, et al. [Sensitivity and specificity of the Patient Health Questionnaire-9 (PHQ-9) among adults from the general population.] *Cad Saude Publica* 2013;29:1533-43. doi: 10.1590/0102-311x00144612

A98. Stafford L, Berk M, Jackson HJ. Validity of the Hospital Anxiety and Depression Scale and Patient Health Questionnaire-9 to screen for depression in patients with coronary artery disease. *Gen Hosp Psychiatry* 2007;29:417-24. doi: 10.1016/j.genhosppsych.2007.06.005

A99. Sung SC, Low CC, Fung DS, et al. Screening for major and minor depression in a multiethnic sample of Asian primary care patients: a comparison of the nine-item Patient Health Questionnaire (PHQ-9) and the 16-item Quick Inventory of Depressive Symptomatology - Self-Report (QIDS-SR16). *Asia Pac Psychiatry* 2013;5:249-58. doi: 10.1111/appy.12101

A100. Suzuki K, Kumei S, Ohhira M, et al. Screening for major depressive disorder with the Patient Health Questionnaire (PHQ-9 and PHQ-2) in an outpatient clinic staffed by primary care physicians in Japan: a case control study. *PloS One* 2015;10:e0119147. doi: 10.1371/journal.pone.0119147

© 2024 Levis B et al. *JAMA Network Open*

A101. van Heyningen T, Honikman S, Tomlinson M, et al. Comparison of mental health screening tools for detecting antenatal depression and anxiety disorders in South African women. *PloS One* 2018;13:e0193697. doi: 10.1371/journal.pone.0193697

A102. van Steenbergen-Weijenburg KM, de Vroege L, Ploeger RR, et al. Validation of the PHQ-9 as a screening instrument for depression in diabetes patients in specialized outpatient clinics. *BMC Health Serv Res* 2010;10:235. doi: 10.1186/1472-6963-10-235

A103. Volker D, Zijlstra-Vlasveld MC, Brouwers EP, et al. Validation of the Patient Health Questionnaire-9 for major depressive disorder in the occupational health setting. *J Occup Rehabil* 2016;26:237-44. doi: 10.1007/s10926-015-9607-0

A104. Wang W, Bian Q, Zhao Y, et al. Reliability and validity of the Chinese version of the Patient Health Questionnaire (PHQ-9) in the general population. *Gen Hosp Psychiatry* 2014;36:539-44. doi: 10.1016/j.genhosppsych.2014.05.021

A105. Zhang Y, Ting R, Lam M, et al. Measuring depressive symptoms using the Patient Health Questionnaire-9 in Hong Kong Chinese subjects with type 2 diabetes. *J Affect Disord* 2013;151:660-66. doi: 10.1016/j.jad.2013.07.01

© 2024 Levis B et al. *JAMA Network Open*
