# Supplementary material for: Data-Driven Cutoff Selection for the Patient Health Questionnaire-9 Depression Screening Tool
Source: JAMA Netw Open. 2024 Nov 22;7(11):e2429630. doi: 10.1001/jamanetworkopen.2024.29630 (PMC11584932; doi:10.1001/jamanetworkopen.2024.29630)
Supplement: Supplement 2. — Data Sharing Statement [file jamanetwopen-e2429630-s002.pdf]

Data Sharing Statement

Levis. Optional Cutoffs and Accuracy Estimates of the Patient Health Questionnaire-9 Depression Screening Tool. *JAMA Netw Open*. Published November 14, 2024. doi:10.1001/jamanetworkopen.2024.29630

Data
Data available: No

Additional Information
Explanation for why data not available: The data are from an individual participant data meta-analysis of 100 primary studies. The data are not publicly available due to ethics requirements and data transfer agreements with principal investigators of the primary studies. Requests to access data should be made to the corresponding author.
